# Circular RNA regulatory network reveals cell–cell crosstalk in acute myeloid leukemia extramedullary infiltration

**DOI:** 10.1186/s12967-018-1726-x

**Published:** 2018-12-17

**Authors:** Chengfang Lv, Lili Sun, Zhibo Guo, Huibo Li, Desheng Kong, Bingqi Xu, Leilei Lin, Tianjiao Liu, Dan Guo, Jin Zhou, Yinghua Li

**Affiliations:** 1 Department of Hematology, The First Affiliated Hospital, Harbin Medical University, 23 Youzheng Street, Nan Gang District, Harbin, 150001 China; 2 Department of Hematology, The Fourth Affiliated Hospital, Harbin Medical University, Harbin, 150001 China

**Keywords:** Acute myeloid leukemia, Extramedullary infiltration, Circular RNAs/circRNAs, Regulatory network, Cell–cell crosstalk, CMap

## Abstract

**Background:**

Acute myeloid leukemia can develop as myoblasts infiltrate into organs and tissues anywhere other than the bone marrow, which called extramedullary infiltration (EMI), indicating a poor prognosis. Circular RNAs (circRNAs) are a novel class of non-coding RNAs that feature covalently closed continuous loops, suggesting their potential as micro RNA (miRNA) “sponges” that can participate in biological processes and pathogenesis. However, investigations on circRNAs in EMI were conducted rarely. In this study, the overall alterations of circRNAs and their regulatory network between EMI and non-EMI AML were delineated.

**Methods:**

CircRNA and whole genome microarrays derived from EMI and non-EMI AML bone marrow mononuclear cells were carried out. Functional analysis was performed via Gene Ontology and KEGG test methods. The speculated functional roles of circRNAs were based on mRNAs and predicted miRNAs that played intermediate roles. Integrated bioinformatic analysis was conducted to further characterize the circRNA/miRNA/mRNA regulatory network and identify the functions of distinct circRNAs. The Cancer Genome Atlas (TCGA) data were acquired to evaluate the poor prognosis of distinct target genes of circRNAs. Reverse transcription-quantitative polymerase chain reaction was conducted to identify the expression of has_circRNA_0004520. Connectivity map (CMap) analysis was further performed to predict potential therapeutic agents for EMI.

**Results:**

253 circRNAs and 663 genes were upregulated and 259 circRNAs and 838 genes were downregulated in EMI compared to non-EMI AML samples. GO pathways were enriched in progress including cell adhesion (GO:0030155; GO:0007155), migration (GO:0016477; GO:0030334), signal transduction (GO:0009966; GO:0007165) and cell–cell communication. Overlapping circRNAs envolved in pathways related to regulate cell–cell crosstalk, 17 circRNAs were chosen based on their putative roles. 7 target genes of 17 circRNAs (LRRK1, PLXNB2, OLFML2A, LYPD5, APOL3, ZNF511, and ASB2) indicated a poor prognosis, while overexpression of PAPLN and NRXN3 indicated a better one based on data from TCGA. LY-294002, trichostatin A and SB-202190 were identified as therapeutic candidates for EMI by the CMap analysis.

**Conclusion:**

Taken together, this study reveals the overall alterations of circRNA and mRNA involved in EMI and suggests potential circRNAs may act as biomarkers and targets for early diagnosis and treatment of EMI.

**Electronic supplementary material:**

The online version of this article (10.1186/s12967-018-1726-x) contains supplementary material, which is available to authorized users.

## Background

Acute myeloid leukemia (AML) is one of the most common hematologic malignancies that aggressive myeloid blasts derived from bone marrow (BM) have disseminated into the peripheral blood (PB), leading to the accumulation of myeloblasts in both the BM and PB [1]. Remarkably, BM-derived blasts may also represent extramedullary infiltration (EMI) in areas other than the BM, including cutis injury, myeloid sarcoma, lymphoid infiltration and central nervous system (CNS) infiltration [2]. EMI may be present at the initial diagnosis of AML, during relapse, or even when patients achieve a complete remission in the BM. EMI predicts a poor outcome of AML due to the destruction of important organs (e.g., in the CNS) and indicates that refractory and relapse leukemia are likely to develop [3]. EMI is often misdiagnosed as inflammation, lymphoma or melanoma [4].

Current studies on the molecular and cellular manifestations of EMI center on cytogenetic backgrounds [1, 5], gene mutations [6], cluster of differentiation (CD) markers and significant non-coding RNAs [7]. Early diagnosis before the appearance of clinical manifestations and interventional treatment may delay disease progression. Furthermore, clarifying the alterations of biological function underlying EMI may provide support to overcome the disease.

Circular RNAs (circRNAs) were considered outcomes of a “splicing error” for decades [8]. However, with the development of high throughput analysis, the functions of circRNAs are being identified. Their specific covalently closed loop structure facilitates their resistance to RNase R, which leads to a half-life of approximately 24 h, compared to the 4–6 h half-life of mRNA at 4 °C [9]. Additionally, circRNAs exhibit tissue and temporal expression specificity and participate in multiple biological processes and human diseases. Emerging evidence reveals that circRNAs can act as miRNA “sponges” [10], interact with RNA binding proteins [11], and regulate parental gene expression, and some can even be translated [12]. Deregulated circRNAs exhibit close relationships with hematological malignancies, especially in AML, where circRNAs are derived from the NMP1 gene [13], and fusion-circRNA (f-circRNA) is derived from the fusion gene MLL-AF9 [14]. However, neither circRNA analysis nor microarray analysis of EMI in AML have been previously conducted.

This study aims to provide an overview of circRNA profiles in EMI to identify biomarkers to aid early diagnosis and treatment. A circRNA/miRNA/mRNA regulatory network was constructed and subjected to a pathway analysis, which revealed that cell–cell interactions are instrumental in EMI; a connectivity map (CMap) analysis was performed based on mRNA dysregulated in the whole regulatory network that indicates three potential therapeutic agents (LY-294002, trichostatin A and SB-202190) for EMI; these results improve our understanding of the cellular progression and pathogenesis of EMI.

## Materials and methods

### Samples

Twelve samples from 4 matched AML patients with and without EMI and healthy volunteers were used in our study. The study was approved by the Ethics Committee of the First Affiliated Hospital of Harbin Medical University. Informed consent was obtained in accordance with the Declaration of Helsinki. Patients with EMI found at diagnosis or at relapse were included, and the clinical features included leukemic cutis, imaging evidence of sarcoma, lymphadenectasis, or manifestations of other types of organ destruction, together with morphological characteristics of myoblast infiltration. The diagnosis was established using the WHO diagnostic criteria [15]. Samples of AML patients without EMI were collected during diagnostic biopsy. The detailed patient information is described in Table 1.

**Table 1:** Clinical characters of patients with or without EMI subject to circRNA expression profiles

Case	Age	FAB	Karyotype	Gene mutation	Group
1	68	M5a	Normal	RUNX1; ASXL1; TET2; IDH2	EMI
2	49	M5a	Normal	IDH2; STAG2; NRAS	EMI
3	35	M2b	46,XX,der(1)ins(21;1)(q22;q21,q42)der(8)t(8;21)(q22;q22),der(21)t(8;21)ins(21;1)	KIT	EMI
4	64	M4	Normal	N/A	EMI
5	42	M5b	Normal	NMP1; DNMT3A; PTPN11; SMC3	Non-EMI
6	23	M2a	Normal	None	Non-EMI
7	48	M1	Normal	None	Non-EMI
8	31	M2a	Normal	None	Non-EMI
9	32	N/A	N/A	N/A	Healthy volunteers
10	28	N/A	N/A	N/A	Healthy volunteers
11	37	N/A	N/A	N/A	Healthy volunteers
12	41	N/A	N/A	N/A	Healthy volunteers

*FAB* French–American–British classification system classifications, *N/A* not available, *EMI* extramedullary infiltration

### Cell separation

Bone marrow mononuclear cells (BMMCs) from AML patients and healthy volunteers were separated through density gradient centrifugation by Ficoll-Hypaque (Solarbio, Beijing, China) as previously reported [16, 17].

### Total RNA extraction

The total RNA was isolated from the samples of both groups using TRIzol (Invitrogen, CA, USA) and a miRNeasy mini kit (QIAGEN, CA, USA) according to the manufacturer’s instructions. The RNA quality and quantity were accurately measured using a NanoDrop spectrophotometer (ND-1000, Nanodrop Technologies, DE, USA). The RNA integrity was assessed by gel electrophoresis.

### Whole genome microarray

The Human Whole Genome Oligo Microarrays (one-color) (Agilent, CA, USA), which covers 44,000 probes detecting approximately 27,958 genes obtained from NCBI and other authoritative data sources. Total RNA was then subjected to reverse transcription and converted to double-strand cDNA (ds-cDNA) using an Invitrogen SuperScript ds-cDNA synthesis kit with oligo dT primers. The ds-cDNA was then purified and labeled with Cy3 using the NimbleGen One-Color DNA labeling kit in accordance with the NimbleGen Gene Expression Analysis Procedure (NimbleGen Systems, Inc., Madison, WI, USA). Thus, the Cy3 labeled-ds-cDNA was hybridized with the microarrays, which were then washed with the Wash Buffer kit (NimbleGen Systems, Inc., Madison, WI, USA).

### CircRNA microarray

CircRNAs from the 12 samples were detected by the Arraystar Human circRNA Array V2 (8 × 15 K, Arraystar, Rockville, MD, USA) including 15,000 probes detecting 13,617 circRNAs ever obtained from circbase and other databases. Total RNA from each sample was quantified using the NanoDrop ND-1000 (Thermo Scientific, DE, USA). The sample preparation and microarray hybridization were performed based on the Arraystar’s standard protocols. Briefly, total RNAs were digested with Rnase R (Epicentre, WI, USA) to remove linear RNAs and enrich circular RNAs. Then, the enriched circular RNAs were amplified and transcribed into fluorescent cRNA utilizing a random priming method (Arraystar Super RNA Labeling Kit; Arraystar, Rockville, MD, USA). The labeled cRNAs were hybridized onto the Arraystar Human circRNA Array V2. After having washed the slides, the arrays were scanned by the Agilent Scanner G2505C.

### Microarray data analysis

Agilent feature extraction software (version 11.0.1.1) was used to analyze acquired both genome expression and circRNA array images. Quantile normalization and subsequent data processing were performed using the R software limma package. Differentially expressed genes and circRNAs with statistical significance between two groups were identified through Volcano Plot filtering. Differentially expressed circRNAs between two samples were identified through Fold Change filtering. Hierarchical Clustering was performed to show the distinguishable circRNAs expression pattern among samples.

Microarray data were uploaded into the gene expression omnibus (GEO) database (https://www.ncbi.nlm.nih.gov/geo/) (accession no. GSE116616, GSE116617).

### GO and KEGG enriched pathway analysis

To explore the underlying pathways and biological processes in EMI, we conducted GO and KEGG pathway analyses based on all differentially expressed mRNAs using the Database for Annotation Visualization and Integrated Discovery (DAVID version 6.8; https://david.ncifcrf.gov/) [18] and the latest KEGG database (http://www.genome.jp/kegg/) [19].

### Target miRNA and mRNA prediction and regulatory network construction

MiRNAs targeted by circRNAs were predicted using TagetScan [20] and miRanda [21] (Threshold: targetscan ≤ 0.05, sitecoverage ≥ 3). miRanda was used for MRE identification and evaluation, and TargetScan was used to supplement the MRE evaluation. Putative miRNAs were listed based on competitive binding ability, and the top 5 miRNAs were selected for further mRNA predictions.

mRNA was predicted by TargetScan, microT and Tarbase on the DIANA-miRPath v.3 platform [22] (Threshold: TargetScan: − 0.4, microT:0.8), and only the target mRNAs presented more than 2 times among the 3 databases were considered target genes of the given miRNAs. The targeted mRNAs were then overlapped with our whole genome microarray data.

A circRNA/miRNA/mRNA regulatory network was then constructed. Cytoscape software (version 3.6.0; http://www.cytoscape.org) was used to delineate the EMI-related gene regulatory network [23].

### Validation the expression of hsa_circRNA_0004520 by reverse transcription-quantitative polymerase chain reaction

RT-PCR was conducted on ViiA 7 Real-time PCR System (Applied Biosystems, Foster City, CA, USA) based on the above 4 pairs of EMI and non-EMI AML samples. Hsa_circRNA_0004520 was amplified using the specific primers shown in Table 2. The PCR reaction system with 10 μl volume consisted of 2 μl cDNA, 0.5 μl of primers (10 μM), 5 μl of 2× Master Mix and 2 μl of H_2_O. The amplification conditions of qRT-PCR reaction was carried out at 95 °Cfor 10 min, followed by 40 cycles at 95 °C for 10 s, 60 °C for 60 s to collect fluorescence, and finally followed by the melting program at 95 °C for 10 s, 60 °C for 60 s,95 °C for 15 s, and 60 °C to 99 °C slowly.

**Table 2:** Primer sequences of circRNA and mRNA measured by qRT-PCR

ID	Primer sequence		Product length (bp)
	Forward (5′–3′)	Reverse (5′–3′)	
β-actin(H)	GTGGCCGAGGACTTTGATTG	CCTGTAACAACGCATCTCATATT	73
hsa_circRNA_004520	TGAGACCAAGAAGGAGAAAAGAAAA	CTCGCACATCGAAGAGGTCA	145

### TCGA data accession and prognostic analysis

To further validate that EMI-related circRNAs and targeted genes have the potential to predict a poor prognosis in AML patients, clinical and RNA-seq data were acquired from TCGA database via LinkedOmics (http://www.linkedomics.org/) [24]. Thirty-five genes targeted by 17 candidate circRNAs were further analyzed according to the protocol, and the parameters were set as follows: Cancer Type: acute myeloid leukemia (AML); RNA-seq based on HiSeq RNA conducted on 01/28/2016 was selected; the dataset for an ethnicity of not Hispanic or Latino was chosen; the target dataset was a clinical dataset conducted on 01/28/2016. Statistical analysis was performed by a non-parametric test, with a value of p ≤ 0.05 considered statistically significant.

## CMap analysis

Differentially expressed genes involved in the circRNA/miRNA/mRNA regulatory network were listed into up- and down-regulated tags and then were uploaded into the CMap web tool (https://portals.broadinstitute.org). Matches between the signatures of interest and chemicals from CMap were assessed through a connectivity score from −1 to 1 that a positive score indicates the stimulative effect of compound on the RNAs; while a negative score implicates a inhibiting effect of a compound [25].

## Results

### CircRNA and differential gene expression in EMI and non-EMI AML BM samples

We examined 4 matched samples each of AML patients with or without EMI and healthy adults using circRNA (Arraystar Human circRNA Array V2, 8 × 15 K, Arraystar, Rockville, MD, USA) and whole genome microarrays (Agilent, CA, USA) at the Department of Hematology of The First Affiliated Hospital of Harbin Medical University.

**Fig. 1:**
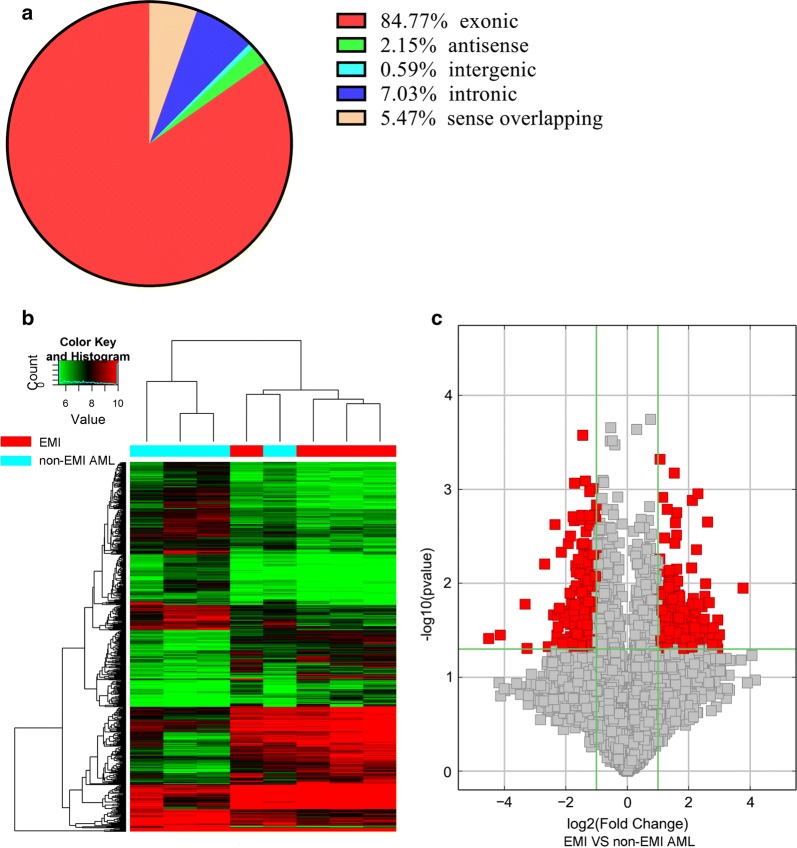
Microarray information of differential circRNA profiles from EMI and non-EMI AML samples. **a** The percentage of significantly differentially expressed circRNAs derived from different genomic loci (exonic, intronic, antisense, intergenic, and sense overlapping). **b** Volcano plot. X-axis: the fold change expressed as log2; Y-axis: p-value expressed as − log10. The vertical green lines correspond to 2.0-fold up- and downregulation, and the horizontal green line represents a p-value of 0.05. The red points in the plot represent circRNAs that were significantly differentially expressed. **c** Hierarchical clustering of circRNA expression data. Samples are classified into different groups based on their expression levels, revealing distinguishable circRNA expression profiling among the EMI and non-EMI AML samples

**Fig. 2:**
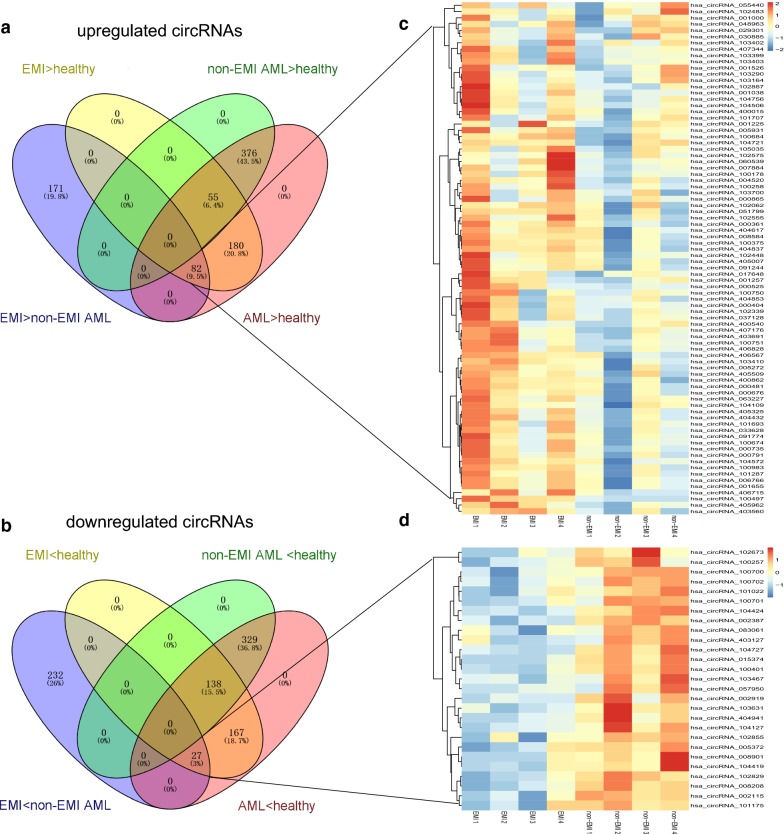
Heatmap of circRNA in EMI and non-EMI AML samples. The overlap patterns were significantly different for circRNAs in the EMI and non-EMI AML groups versus those of healthy volunteers. There were 253 significantly upregulated (**a**) and 259 downregulated (**b**) circRNAs in the EMI versus the non-EMI AML group (purple area). Eighty-two significantly upregulated (**a**) and 27 downregulated (**b**) circRNAs were found among all AML patients (EMI and non-EMI AML) versus healthy volunteers. Finally, 171 significantly upregulated (**a**) and 232 downregulated (**b**) circRNAs were likely responsible for EMI after exclusion of those responsible for AML

CircRNAs and mRNAs with fold changes ≥ 2.0 and p-values ≤ 0.05 were considered significantly differentially expressed. Two hundred fifty-three circRNAs and 663 genes were upregulated, while 259 circRNAs and 838 genes were downregulated in AML patients with EMI compared to those without EMI (Additional file 1: Tables S1 and Additional file 2: Table S2). Of the differentially expressed circRNAs detected, 84.77% were covered in the exon of the genome (Fig. 1a). Hierarchical clustering was performed between of the AML with and without EMI groups to demonstrate the circRNA expression patterns (Fig. 1b, c). Furthermore, clustering overlapping was conducted to identify the circRNAs likely responsible for EMI. There were 353 upregulated circRNAs in the EMI versus non-EMI AML groups, which further overlapped with circRNAs upregulated in the AML with EMI versus the healthy groups. Finally, 82 upregulated and 27 downregulated circRNAs were considered to have closer relationships with EMI (Fig. 2a–d).

### Pathway enrichment of differentially expressed genes in EMI based on GO and KEGG analysis

To clarify the biological processes related to EMI, Gene Ontology (GO) analysis and Kyoto Encyclopedia of Genes and Genomics (KEGG) pathway enrichment were performed on all 663 upregulated and 838 downregulated genes aberrantly expressed in the EMI group.

**Fig. 3:**
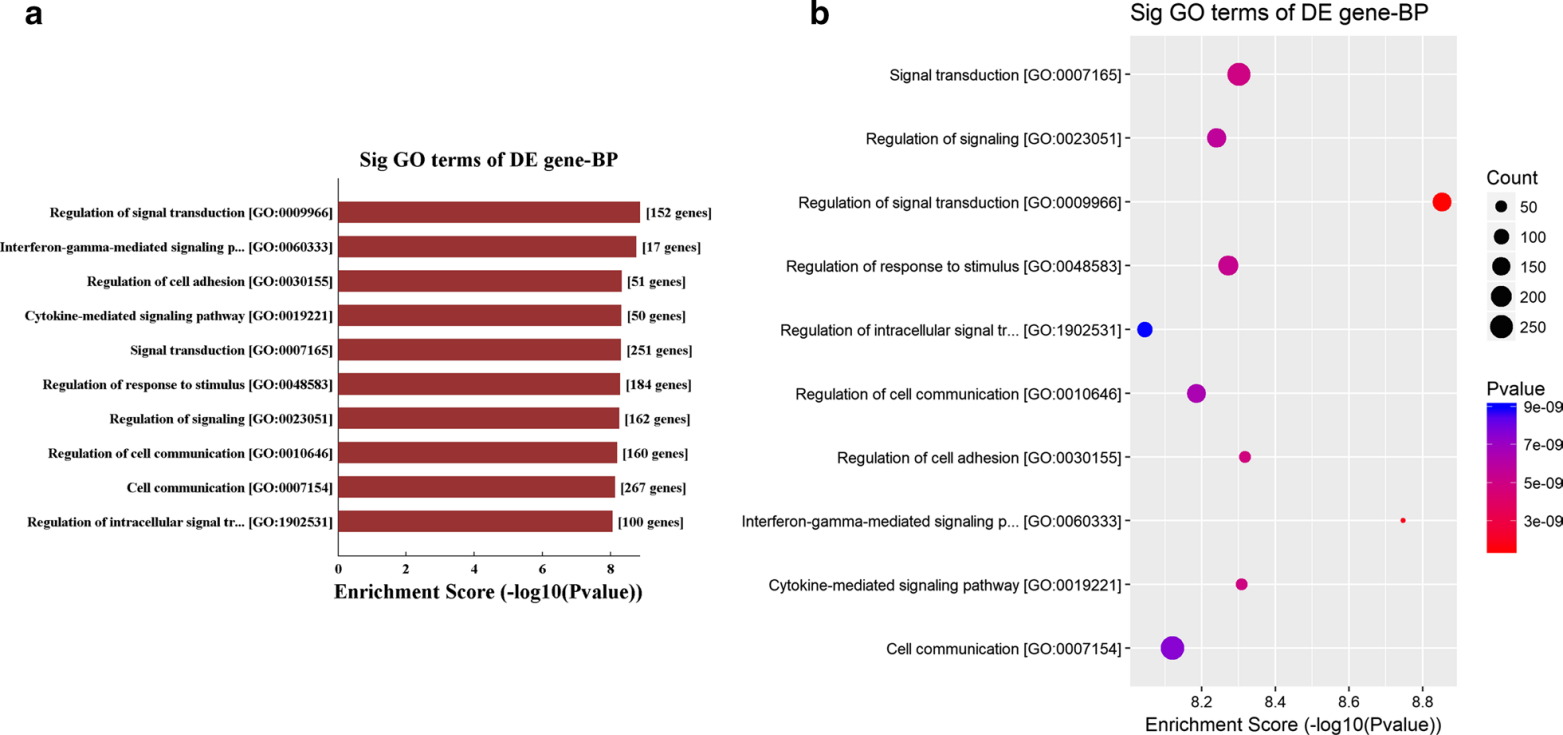
Pathway analysis of EMI-related circRNAs and genes. **a**, **b** GO analysis of all 663 upregulated genes in EMI versus non-EMI AML samples. The activated biological processes related to EMI were principally involved in cell–cell crosstalk, including cell adhesion (GO:0030155; GO:0007155), migration (GO:0016477; GO:0030334), signal transduction (GO:0009966; GO:0007165) and cell–cell communication (GO:0010646; 0007154). The X-axis represents the ratio of enriched differential genes (**a**, **b**). The Y-axis shows the names of process that were significantly enriched (**a**, **b**). The size of each node represents the number of enriched differential genes, and the p-values are indicated by color changes from red to purple (**b**). Downregulated genes were not associated with specific EMI-related processes

The GO analysis results revealed that these upregulated genes participated in vital biological processes including cell adhesion (GO:0030155; GO:0007155), migration (GO:0016477; GO:0030334), signal transduction (GO:0009966; GO:0007165) and cell–cell communication (GO:0010646; 0007154) (Fig. 3a, b, Additional file 3: Table S3 and Additional file 4: Table S4), which is consistent with current reports on the relationship between EMI and the microenvironment [26].

KEGG enriched pathway analysis indicated that several significative pathways might be aberrantly activated in response to EMI, including the RAP1 signaling pathway (hsa04015), Cell adhesion molecules (hsa04514) and other immunology related pathways (Additional file 5: Table S5 and Additional file 6: Table S6).

### Construction and visualization of the circRNA/miRNA/mRNA regulatory network

Given the potential regulatory roles of circRNAs on miRNA “sponges”, two algorithms that focused on miRNA response elements (MREs) between circRNA and miRNA (miRanda and TargetScan) were used to predict miRNAs related to EMI. To further associate circRNAs with biological processes, we delineated the regulation of circRNAs and expressed genes indirectly, i.e., the circRNA/miRNA/mRNA network. We included the top 5 miRNAs regulated by circRNAs, and then searched the list of miRNAs in both the miRTarBase and TargetScan databases to obtain putative target genes. Finally, we overlapped the predicted target mRNAs with our whole genome microarray profiles. A regulatory network involving circRNAs/miRNAs/genes was constructed using Cytoscape software (version 3.6.0; http://www.cytoscape.org) Additional file 7: Figure S1), and the prediction of functional roles of circRNA was based on the downstream genes.

### Seventeen circRNAs were associated with vital processes in EMI

**Fig. 4:**
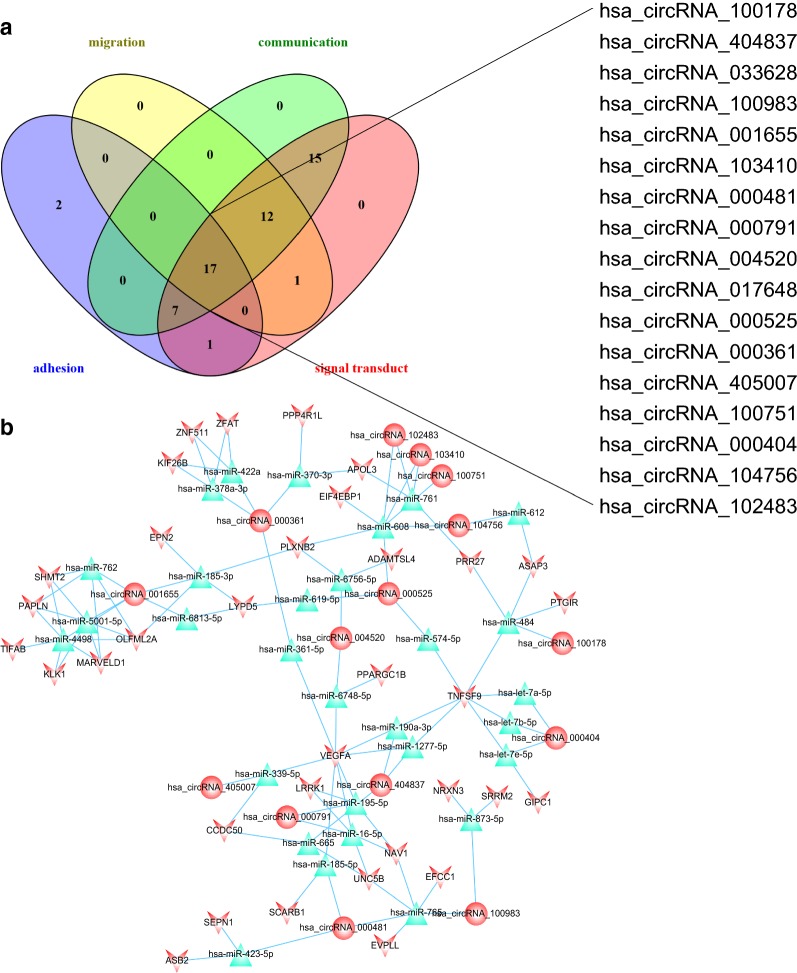
circRNAs involved in vital processes in EMI. **a** circRNAs related to cell–cell crosstalk were further overlapped to identify circRNAs that act as “kernels” in EMI. Seventeen circRNAs had the potential to regulate all 4 processes including cell migration, cell–cell communication, signal transduction, and cell adhesion. **b** The 17 “kernel” circRNAs and their target miRNAs and genes were finally delineated into a regulatory network. Round nodes represent circRNAs, V nodes represent differentially expressed genes, and red represents upregulated genes. Blue triangles represent predicted miRNAs. Lines indicate a regulatory relationship

**Fig. 5:**
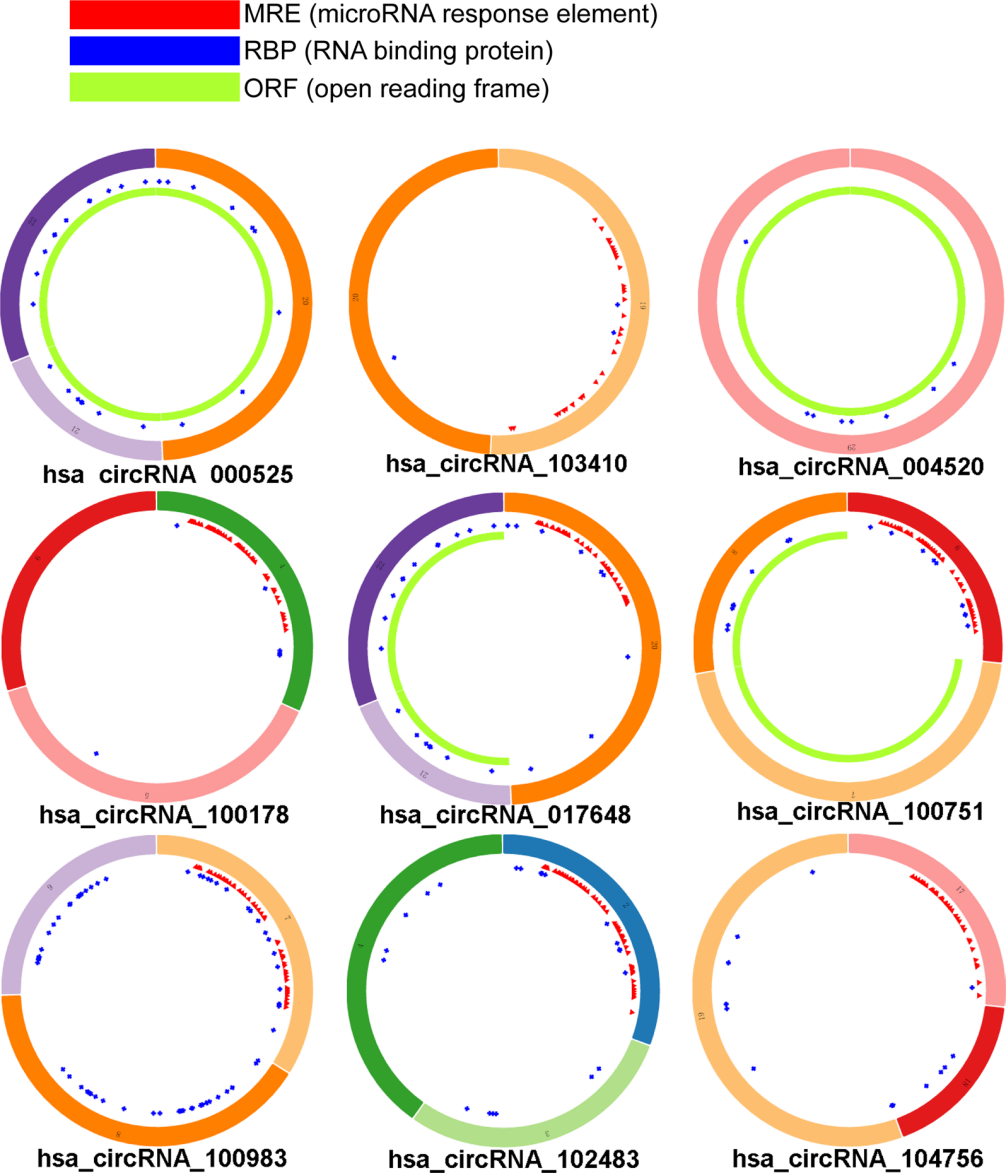
Structural patterns of the 9 circRNAs by the Cancer-Specific CircRNA. 9 of all 17 key circRNAs are available in the database

To explore the candidate circRNAs that may play vital roles in EMI of AML, 82 upregulated and 27 downregulated circRNAs were selected for further analysis after overlapping. We then eliminated candidate circRNAs based on their associated biological processes. To determine which circRNAs were involved in the regulation of cell–cell interactions, genes related to these processes were retained. Thirty circRNAs were responsible for cell migration, 51 were responsible for cell–cell communication, 53 were responsible for signal transduction, and 27 were responsible for cell adhesion. CircRNAs involved in the processes mentioned above were further overlapped to identify the “kernel” circRNA. After excluding “irrelevant” genes, miRNAs and circRNAs, 17 circRNAs likely to play important roles in EMI remained, and the network of circRNAs related to EMI was diagrammed (Table 3, Fig. 4). And the structure of 9 circRNAs (8 not available) were described in Fig. 5 based on the data from Cancer Specific CircRNA (CSCD, http://gb.whu.edu.cn/CSCD/) [27].

**Table 3:** Essential characters of the 17 key DECs responsible for EMI

CircRNA	Alias	CircRNA type	Chrom	Strand	txStart	txEnd	Best transcript	Gene symbol
hsa_circRNA_100178	hsa_circ_0008844	Exonic	chr1	+	40,422,758	40,424,497	NM_032793	MFSD2A
hsa_circRNA_404837	–	Intronic	chr11	−	3,777,940	3,778,768	ENST00000324932	NUP98
hsa_circRNA_033628	hsa_circ_0033628	Exonic	chr14	+	105,953,535	105,955,124	NM_001311	CRIP1
hsa_circRNA_100983	hsa_circ_0024766	Exonic	chr11	+	125,472,197	125,474,142	NM_152713	STT3A
hsa_circRNA_001655	hsa_circ_0001655	Intergenic	chr6	−	156,468,903	156,489,628	–	–
hsa_circRNA_103410	hsa_circ_0003266	Exonic	chr3	−	66,501,982	66,512,933	NM_015541	LRIG1
hsa_circRNA_000481	hsa_circ_0001828	Intronic	chr8	+	142,139,086	142,139,265	ENST00000262585	DENND3
hsa_circRNA_000791	hsa_circ_0000282	Intronic	chr11	+	28,250,417	28,255,132	ENST00000303459	METTL15
hsa_circRNA_004520	hsa_circ_0004520	Sense overlapping	chr9	−	136,790,964	136,804,341	NM_003371	VAV2
hsa_circRNA_017648	hsa_circ_0017648	Exonic	chr10	−	7,409,610	7,423,911	NM_001018039	SFMBT2
hsa_circRNA_000525	hsa_circ_0000212	Sense overlapping	chr10	−	7,405,839	7,423,911	NM_001018039	SFMBT2
hsa_circRNA_000361	hsa_circ_0001275	Antisense	chr3	−	17,059,499	17,059,748	NM_015184	PLCL2
hsa_circRNA_405007	–	Intronic	chr12	−	53,468,590	53,468,877	ENST00000301463	SPRYD3
hsa_circRNA_100751	hsa_circ_0020934	Exonic	chr11	+	4,076,755	4,080,626	NM_003156	STIM1
hsa_circRNA_000404	hsa_circ_0001477	Antisense	chr5	+	39,073,734	39,073,896	NM_152756	RICTOR
hsa_circRNA_104756	hsa_circ_0086694	Exonic	chr9	−	33,932,559	33,933,626	NM_018449	UBAP2
hsa_circRNA_102483	hsa_circ_0049998	Exonic	chr19	+	17,626,981	17,628,659	NM_012088	PGLS

### Validation of the expression of hsa_circRNA_0004520

**Fig. 6:**
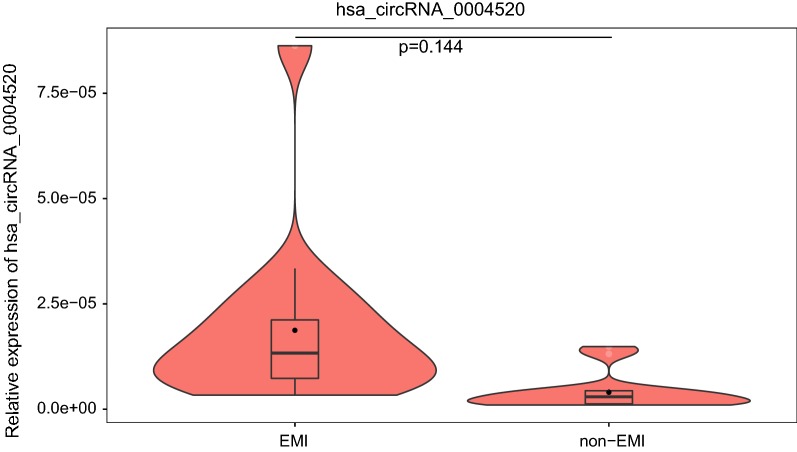
Violin plots for the expression of hsa_circRNA_0004520 in EMI and non-EMI AML samples by RT-PCR. The expression of hsa_circRNA_0004520 is upregulated by 4.38-fold with a p value of 0.144. *EMI* extramedullary infiltration

RT-PCR was conducted in 4 pairs of EMI and non-EMI AML BMMCs to validate the expression of key circRNAs. Hsa_circRNA_0004520 was chosen for our interest on its target genes: PLXNB2 and VEGFA. The expression of hsa_circRNA_0004520 was upregulated by 4.38-fold (p = 0.144) (Fig. 6).

### Validation of the prognostic value of 17 circRNAs that target genes involved in AML

**Fig. 7:**
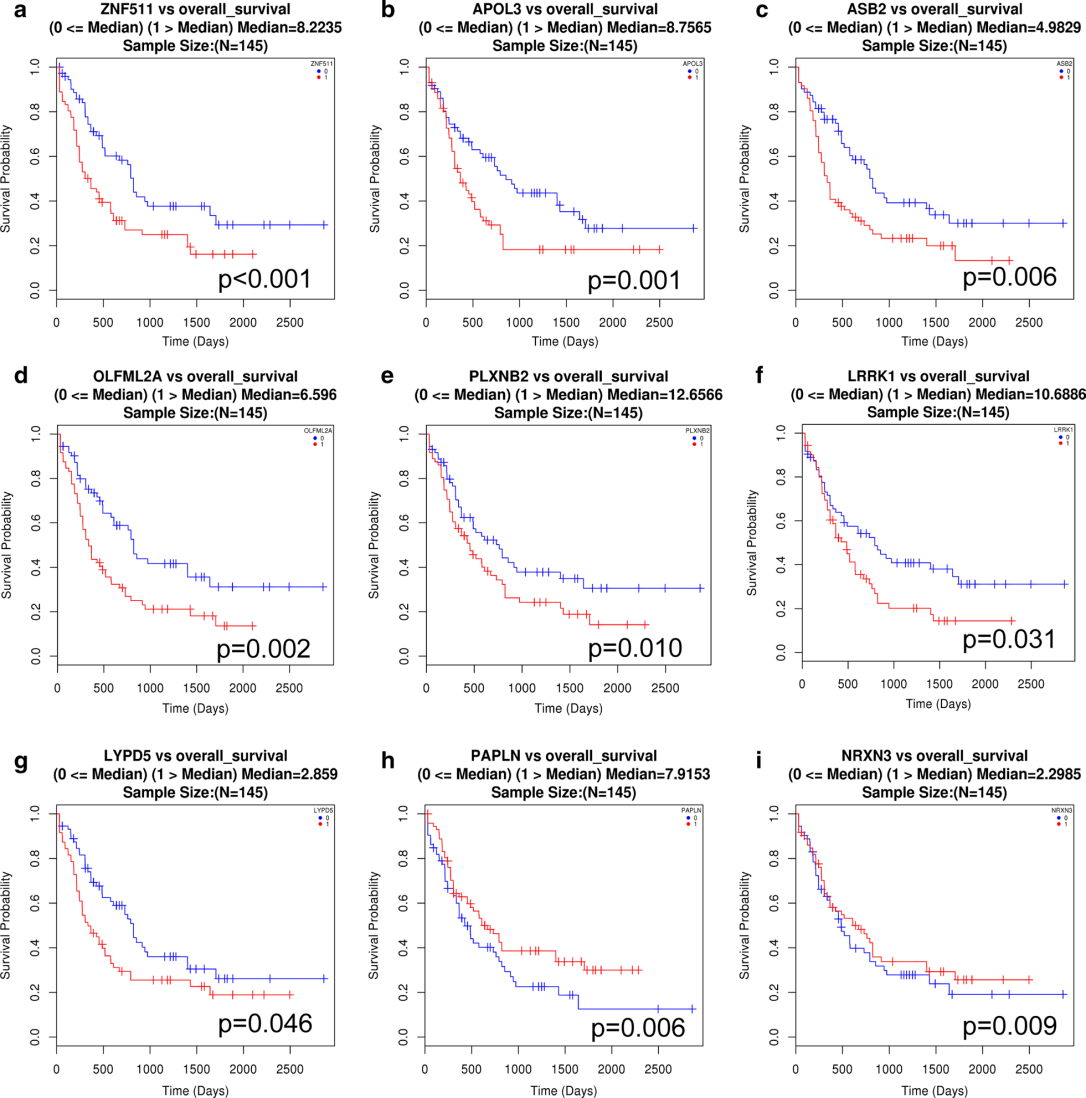
Data from TCGA showing that most mRNAs targeted by the 17 circRNAs are significantly correlated with poor overall survival of AML patients. Overall survival curve of AML patients grouped according to gene expression level. **a** ZNF511, **b** APOL3, **c** ASB2, **d** OLFML2A, **e** PLXNB2, **f** LRRK1 and **g** LYPD5 were significantly correlated with poor survival of AML patients, while **h** PALPN and **i** NRXN3 were correlated with improved survival. Red lines indicate gene expression above the median in AML patients, and blue lines indicate gene expression below the median

To validate the prognostic value of circRNAs related to EMI, we obtained access to the Cancer Genome Atlas (TCGA) database to assess the relationship between the target genes of the 17 circRNAs and the prognosis in AML patients because EMI is associated with a poorer prognosis than non-EMI AML. A non-parametric test was applied to clinical and RNA-seq data, and a value of p ≤ 0.05 was considered a statistically significant difference. AML with overexpression of 7 genes (LRRK1, PLXNB2, OLFML2A, LYPD5, APOL3, ZNF511, and ASB2) indicated a poor prognosis, while overexpression of PAPLN and NRXN3 indicated a better prognosis (Fig. 7).

The data obtained from TCGA suggested the potential roles of circRNA and differentially expressed genes in the AML prognosis and further indicated that they might play crucial roles in EMI. However, this finding should be confirmed in further investigations.

### Identification of three bioactive compounds for the treatment of EMI based on CMap analysis

**Fig. 8:**
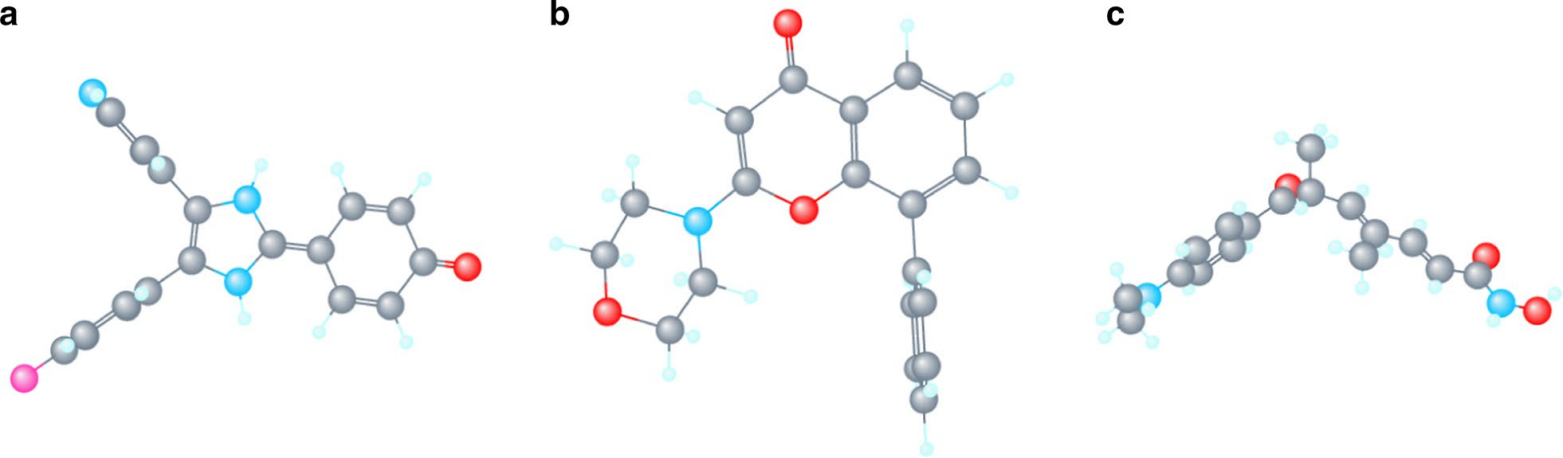
3D-structure of three candidates for EMI by CMap analysis. **a** Trichostatin A (TSA), **b** LY-294002, **c** SB-202190

All differentially expressed mRNA in the circRNA/miRNA/mRNA regulatory network were loaded into the CMap web tool. Following the signature query, three compounds with potential anti-tumor effect, LY-294002, trichostatin A (TSA) and SB-202190, i.e. with the highest negative enrichment score were determined as the potential therapeutic agents for EMI (Table 4). The 3D chemical structures of the three compounds obtained from NCBI are presented in Fig. 8.

**Table 4:** Compounds identified as potential treatment options for EMI based on CMap analysis

CMap names	Enrichment score	Dose (µM)	Cell	Up scores	Down scores
LY-294002	− 0.969	10	PC3	− 0.228	0.284
LY-294002	− 0.968	10	PC3	− 0.307	0.205
LY-294002	− 0.884	10	HL60	− 0.227	0.24
LY-294002	− 0.859	10	HL60	− 0.252	0.202
Trichostatin A	− 0.847	1	HL60	− 0.229	0.219
LY-294002	− 0.833	10	HL60	− 0.319	0.122
LY-294002	− 0.809	10	HL60	− 0.212	0.215
SB-202190	− 0.801	1	MCF7	− 0.149	0.275

*CMap* connectivity map

## Discussion

In this study, we delineated a circRNA/miRNA/gene regulatory network related to EMI using microarray and bioinformatic tools for the first time. The altered genes are downstream in the regulatory network, and the functions were predicted via bioinformatics methods. MiRNAs were used to link mRNAs and circRNAs to predict their function in EMI pathogenesis. CMap analysis revealed LY-294002, trichostatin A (TSA) and SB-202190 to be treatment candidates for EMI based on dysregulated mRNAs. We explored potential biological processes and pathogenesis and predicted conceivable roles for circRNA throughout the EMI network.

EMI is relatively common in AML, with myeloid sarcoma appearing in nearly 1.4–9% of AML patients [28], and leukemic cutis present in approximately 15% of patients. It has been estimated that EMI occurs in 18–25% of childhood AML cases [29]. EMI exhibits a close relationship with relapse and refractory AML. Strikingly, after patients have undergone allo-hematopoietic stem cell transplantation (HSCT), EMI may still appear or even worsen [30].

Previous studies have investigated cytogenetic factors in EMI, such as alterations in chromosome 8 alteration, FLT3-ITD and NPM1 mutations. As the application of high throughput technology has increased, the roles of circRNAs are being clarified. Based on the circRNA/miRNA/mRNA regulatory network, the functions of circRNA in EMI were assessed, which primarily involved cell adhesion, migration, signal transduction and cell–cell communication. Previous reports on EMI have also considered cell–cell communication to play an important role in EMI, with leukemic cells subverting normal interactions and converting a normal niche into a neoplastic niche [31]. circRNAs responsible for multiple processes were thought to play an even more important role. After overlapping of four processes, 17 circRNAs in our study were identified. Due to the interesting targets (PLXNB2 and VEGFA, which may take part in angiogenesis pathways) of has_circRNA-0004520, RT-PCR was performed to validate its expression, and the function needs to be identified in our further investigation. Clinical and RNA-seq data were obtained from TCGA to further evaluate the prognostic predictive ability of EMI-related genes and circRNAs. Prognostic data of 34 in 35 genes targeted by all 17 circRNAs were obtained, of which 9 genes seemed to have a significant prediction. The high expression of 7 genes (ZNF511, APOL3, ASB2, OLFML2A, PLXNB2, LRRK1 and LYPD5, i.e.) predicted a worse outcome. These AML patients might suffer from EMI or molecular alterations before clinical symptoms, and it is our aim to find a biomarker before clinical manifestation arises. Interestingly, 2 genes (PALPN and NRXN3) seemed to exhibit a protective effect against EMI thus prolong the overall survival, which needs to be investigated. We selected circRNA as a novel biomarker for early diagnosis and treatment, thus hinder the progression of EMI and further efforts to overcome this process.

Interestingly, a CMap analysis based on mRNAs dysregulated in EMI was applied to explore practicable drugs for the treatment of EMI. Drugs on CMap were all available for human use and listed in the Food and Drug Administration. Data on CMap are based on genome-wide transcriptional expression profiles and accurately show the promoting and inhibiting effects on gene expression in transcriptional level. Three chemicals were identified to be options for EMI based on CMap results. LY-294002 is considered to be a conventional specific inhibitor of phosphatidylinositol 3-kinase (PI3K) [32]. PI3K/Akt pathways play important roles in multiple oncogenetic processes. LY-294002 has been applied for solid tumors for decades. While in hematological malignancies, there are not any PI3K inhibitors for clinical application. Recent studies on the resistance of chronic myeloid leukemia or to be a sensitizer for chemotherapy reveal that LY-294002 might work in refractory and relapsed leukemia [33, 34], such as EMI. SB-202190 is inhibitor for p38 MAPK signaling pathway [35]. P38 MAPK pathways may even play opposite effect in diverse tumors. Compared to LY-294002, SB-202190 has more applications in AML. SB-202190 can diminish doxorubicin-induced resistance in K562 cells [36], but promote THP-1 and MV4:11 cells also [37], and the exact effect of SB-202190 in EMI needs to be clarified later. As an inhibitor of histone deacetylases (HDAC), trichostatin A (TSA) has been reported for a potent inducer of tumor cell growth arrest, and apoptosis in many diverse transformed cells and tumor-bearing animals by regulating the expression of tumor suppressor genes [38]. HDAC inhibitors are gradually in use for lymphoma and multiple myeloma in this decade [39, 40]. The use of HDAC inhibitor in our EMI patients also appears a modest curative effect (data not shown).

However, there are limitations in this study. First, EMI is a very complex disease. The mechanisms of different subtypes and organs may be different. A larger and more accurate group of samples is needed to clarify these differences. In addition, only parts of circRNAs function as miRNA “sponges”. Some circRNAs may also regulate parent gene expression and participate in EMI. Additionally, classical post-transcriptional regulation of miRNAs allows them to bind to the 3′ UTR region of mRNAs and thus degrade them, while the targeted relationship still needs to be validated. Subsequent investigations will take this finding into account. Notably, our study focused on RNA levels, and further research on protein levels in cell lines and in vivo models is needed.

## Conclusions

Our research is the first to use microarrays of circRNAs and whole genome expression together with bioinformatic tools to identify a circRNA/miRNA/gene regulatory network of EMI in AML patients. Seventeen circRNAs may act as key regulators of cell–cell crosstalk in EMI, and most of their target genes predict a poor prognosis. LY-294002, trichostatin A (TSA) and SB-202190 are potential treatment candidates for EMI. The unique structure and function makes circRNA an ideal biomarker for early, rapid and accurate diagnosis, which can aid efforts to overcome the disease.

## Additional files

**Additional file 1: Table S1.** The list of dysregulated circRNAs between EMI and non-EMI AML patients.

**Additional file 2: Table S2.** The list of dysregulated genes between EMI and non-EMI AML samples.

**Additional file 3: Table S3.** GO analysis of upregulated genes between EMI and non-EMI AML samples.

**Additional file 4: Table S4.** GO analysis of downregulated genes between EMI and non-EMI AML samples.

**Additional file 5: Table S5.** KEGG pathway enrichment of upregulated genes between EMI and non-EMI AML samples.

**Additional file 6: Table S6.** KEGG pathway enrichment of downregulated genes between EMI and non-EMI AML samples.

**Additional file 7: Figure S1.** Regulatory networks of EMI-related circRNAs, miRNAs and genes.

## Abbreviations

AML: acute myeloid leukemia
BM: bone marrow
PB: peripheral blood
EMI: extramedullary infiltration
circRNA: circular RNA
miRNA: micro RNA
GO: gene ontology
KEGG: Kyoto Encyclopedia of Genes and Genomics
MRE: miRNA response elements
HSCT: hematopoietic stem cell transplantation
CSCD: cancer specific circRNA
TCGA: the cancer genome atlas
ORF: open reading frame
RBP: RNA binding protein

## References

[1] Ohanian M, Faderl S, Ravandi F, Pemmaraju N, Garcia-Manero G, Cortes J, Estrov Z (2013). Is acute myeloid leukemia a liquid tumor?. Int J Cancer.

[2] Stolzel F, Rollig C, Radke J, Mohr B, Platzbecker U, Bornhauser M, Paulus T, Ehninger G, Zophel K, Schaich M (2011). (1)(8)F-FDG-PET/CT for detection of extramedullary acute myeloid leukemia. Haematologica.

[3] Jiang L, Yu G, Meng W, Wang Z, Meng F, Ma W (2013). Overexpression of amyloid precursor protein in acute myeloid leukemia enhances extramedullary infiltration by MMP-2. Tumour Biol.

[4] Byrd JC, Edenfield WJ, Shields DJ, Dawson NA (1995). Extramedullary myeloid cell tumors in acute nonlymphocytic leukemia: a clinical review. J Clin Oncol.

[5] Tirado CA, Chen W, Valdez F, Karandikar N, Arbini A, Acevedo I, Garcia R, Davila O, Smart RL, Matthews E, Kirk A, Collins RH (2010). Unusual presentation of myeloid sarcoma in a case of acute promyelocytic leukemia with a cryptic PML-RARA rearrangement involving multiple sites including the atrium. Cancer Genet Cytogenet.

[6] Port M, Bottcher M, Thol F, Ganser A, Schlenk R, Wasem J, Neumann A, Pouryamout L (2014). Prognostic significance of FLT3 internal tandem duplication, nucleophosmin 1, and CEBPA gene mutations for acute myeloid leukemia patients with normal karyotype and younger than 60 years: a systematic review and meta-analysis. Ann Hematol.

[7] Chang H, Brandwein J, Yi QL, Chun K, Patterson B, Brien B (2004). Extramedullary infiltrates of AML are associated with CD56 expression, 11q23 abnormalities and inferior clinical outcome. Leuk Res.

[8] Hsu MT, Coca-Prados M (1979). Electron microscopic evidence for the circular form of RNA in the cytoplasm of eukaryotic cells. Nature.

[9] Li P, Chen S, Chen H, Mo X, Li T, Shao Y, Xiao B, Guo J (2015). Using circular RNA as a novel type of biomarker in the screening of gastric cancer. Clin Chim Acta.

[10] Hansen TB, Jensen TI, Clausen BH, Bramsen JB, Finsen B, Damgaard CK, Kjems J (2013). Natural RNA circles function as efficient microRNA sponges. Nature.

[11] Du WW, Zhang C, Yang W, Yong T, Awan FM, Yang BB (2017). Identifying and characterizing circRNA–protein interaction. Theranostics.

[12] Li Z, Huang C, Bao C, Chen L, Lin M, Wang X, Zhong G, Yu B, Hu W, Dai L, Zhu P, Chang Z, Wu Q, Zhao Y, Jia Y, Xu P, Liu H, Shan G (2015). Exon-intron circular RNAs regulate transcription in the nucleus. Nat Struct Mol Biol.

[13] Hirsch S, Blatte TJ, Grasedieck S, Cocciardi S, Rouhi A, Jongen-Lavrencic M, Paschka P, Kronke J, Gaidzik VI, Dohner H, Schlenk RF, Kuchenbauer F, Dohner K, Dolnik A, Bullinger L (2017). Circular RNAs of the nucleophosmin (NPM1) gene in acute myeloid leukemia. Haematologica.

[14] Guarnerio J, Bezzi M, Jeong JC, Paffenholz SV, Berry K, Naldini MM, Lo-Coco F, Le Tay Y, Beck AH, Pandolfi PP (2016). Oncogenic role of fusion-circRNAs derived from cancer-associated chromosomal translocations. Cell.

[15] Vardiman JW, Thiele J, Arber DA, Brunning RD, Borowitz MJ, Porwit A, Harris NL, Le Beau MM, Hellstrom-Lindberg E, Tefferi A, Bloomfield CD (2009). The 2008 revision of the World Health Organization (WHO) classification of myeloid neoplasms and acute leukemia: rationale and important changes. Blood.

[16] Zhang Y, Li H, Cao R, Sun L, Wang Y, Fan S, Zhao Y, Kong D, Cui L, Lin L, Wang K, Li Y, Zhou J (2017). Suppression of miR-708 inhibits the Wnt/beta-catenin signaling pathway by activating DKK3 in adult B-all. Oncotarget.

[17] Kong D, Zhao L, Sun L, Fan S, Li H, Zhao Y, Guo Z, Lin L, Cui L, Wang K, Chen W, Zhang Y, Zhou J, Li Y (2018). MYCN is a novel oncogenic target in adult B-ALL that activates the Wnt/beta-catenin pathway by suppressing DKK3. J Cell Mol Med.

[18] da Huang W, Sherman BT, Lempicki RA (2009). Bioinformatics enrichment tools: paths toward the comprehensive functional analysis of large gene lists. Nucleic Acids Res.

[19] Kanehisa M, Goto S, Hattori M, Aoki-Kinoshita KF, Itoh M, Kawashima S, Katayama T, Araki M, Hirakawa M (2006). From genomics to chemical genomics: new developments in KEGG. Nucleic Acids Res.

[20] Fromm B, Billipp T, Peck LE, Johansen M, Tarver JE, King BL, Newcomb JM, Sempere LF, Flatmark K, Hovig E, Peterson KJ (2015). A uniform system for the annotation of vertebrate microRNA genes and the evolution of the human microRNAome. Annu Rev Genet.

[21] Betel D, Wilson M, Gabow A, Marks DS, Sander C (2008). The microRNA.org resource: targets and expression. Nucleic Acids Res..

[22] Vlachos IS, Hatzigeorgiou AG (2017). Functional analysis of miRNAs using the diana tools online suite. Methods Mol Biol.

[23] Shannon P, Markiel A, Ozier O, Baliga NS, Wang JT, Ramage D, Amin N, Schwikowski B, Ideker T (2003). Cytoscape: a software environment for integrated models of biomolecular interaction networks. Genome Res.

[24] Vasaikar SV, Straub P, Wang J, Zhang B (2018). LinkedOmics: analyzing multi-omics data within and across 32 cancer types. Nucleic Acids Res.

[25] Musa A, Ghoraie LS, Zhang SD, Glazko G, Yli-Harja O, Dehmer M, Haibe-Kains B, Emmert-Streib F (2017). A review of connectivity map and computational approaches in pharmacogenomics. Brief Bioinform.

[26] Saki N, Abroun S, Farshdousti Hagh M, Asgharei F (2011). Neoplastic bone marrow niche: hematopoietic and mesenchymal stem cells. Cell J.

[27] Xia S, Feng J, Chen K, Ma Y, Gong J, Cai F, Jin Y, Gao Y, Xia L, Chang H, Wei L, Han L, He C (2018). CSCD: a database for cancer-specific circular RNAs. Nucleic Acids Res.

[28] Bakst RL, Tallman MS, Douer D, Yahalom J (2011). How I treat extramedullary acute myeloid leukemia. Blood.

[29] Johnston DL, Alonzo TA, Gerbing RB, Lange BJ, Woods WG (2012). Superior outcome of pediatric acute myeloid leukemia patients with orbital and CNS myeloid sarcoma: a report from the Children’s oncology group. Pediatr Blood Cancer.

[30] Kogut N, Tsai NC, Thomas SH, Palmer J, Paris T, Murata-Collins J, Forman SJ (2013). Extramedullary relapse following reduced intensity allogeneic hematopoietic cell transplant for adult acute myelogenous leukemia. Leuk Lymphoma.

[31] Saki N, Shahjahani M, Azizidoost S, Khosravi A, Mohammadiasl J (2015). Molecular and cellular aspects of extramedullary manifestations of acute myeloid leukemia. J Cancer Meta Treat..

[32] Vlahos CJ, Matter WF, Hui KY, Brown RF (1994). A specific inhibitor of phosphatidylinositol 3-kinase, 2-(4-morpholinyl)-8-phenyl-4*H*-1-benzopyran-4-one (LY294002). J Biol Chem.

[33] Liu P, Ma D, Yu Z, Zhe N, Ren M, Wang P, Yu M, Huang J, Fang Q, Wang J (2017). Overexpression of heme oxygenase-1 in bone marrow stromal cells promotes microenvironment-mediated imatinib resistance in chronic myeloid leukemia. Biomed Pharmacother.

[34] Yu C, Mao X, Li WX (2005). Inhibition of the PI3K pathway sensitizes fludarabine-induced apoptosis in human leukemic cells through an inactivation of MAPK-dependent pathway. Biochem Biophys Res Commun.

[35] Shanware NP, Williams LM, Bowler MJ, Tibbetts RS (2009). Non-specific in vivo inhibition of CK1 by the pyridinyl imidazole p38 inhibitors SB 203580 and SB 202190. BMB Rep.

[36] Chen Y, Zhao Y, Wang C, Xiao X, Zhou X, Xu G (2012). Inhibition of p38 MAPK diminishes doxorubicin-induced drug resistance associated with P-glycoprotein in human leukemia K562 cells. Med Sci Monit.

[37] Hirosawa M, Nakahara M, Otosaka R, Imoto A, Okazaki T, Takahashi S (2009). The p38 pathway inhibitor SB202190 activates MEK/MAPK to stimulate the growth of leukemia cells. Leuk Res.

[38] Ozaki K, Kishikawa F, Tanaka M, Sakamoto T, Tanimura S, Kohno M (2008). Histone deacetylase inhibitors enhance the chemosensitivity of tumor cells with cross-resistance to a wide range of DNA-damaging drugs. Cancer Sci.

[39] Ji MM, Huang YH, Huang JY, Wang ZF, Fu D, Liu H, Liu F, Leboeuf C, Wang L, Ye J, Lu YM, Janin A, Cheng S, Zhao WL (2018). Histone modifier gene mutations in peripheral T-cell lymphoma not otherwise specified. Haematologica.

[40] He J, Chen Q, Gu H, Chen J, Zhang E, Guo X, Huang X, Yan H, He D, Yang Y, Zhao Y, Wang G, He H, Yi Q, Cai Z (2018). Therapeutic effects of the novel subtype-selective histone deacetylase inhibitor chidamide on myeloma-associated bone disease. Haematologica.

