# Supplementary material for: Circular RNA regulatory network reveals cell–cell crosstalk in acute myeloid leukemia extramedullary infiltration
Source: J Transl Med. 2018 Dec 17;16:361. doi: 10.1186/s12967-018-1726-x (PMC6297994; doi:10.1186/s12967-018-1726-x)
Supplement: Supplementary file 1 — Additional file 1: Table S1. The list of dysregulated circRNAs between EMI and non-EMI AML patients. [file 12967_2018_1726_MOESM1_ESM.docx]

| **Table S1 The list of dysregulated circRNAs between EMI and non-EMI AML patients** | | |
| --- | --- | --- |
| **number of differentially expressed circRNAs(up-/down-reuglated)** | **up-regulated circRNAs** | **Down-regulated circRNAs** |
| 512(253/259) | hsa_circRNA_403560,hsa_circRNA_404837,hsa_circRNA_062557,hsa_circRNA_104573,hsa_circRNA_033628,hsa_circRNA_100983,hsa_circRNA_104168,hsa_circRNA_101015,hsa_circRNA_400862,hsa_circRNA_403691,hsa_circRNA_001655,hsa_circRNA_100684,hsa_circRNA_101287,hsa_circRNA_008584,hsa_circRNA_406828,hsa_circRNA_083776,hsa_circRNA_103410,hsa_circRNA_404432,hsa_circRNA_023461,hsa_circRNA_407176,hsa_circRNA_406567,hsa_circRNA_005054,hsa_circRNA_103923,hsa_circRNA_058191,hsa_circRNA_000481,hsa_circRNA_405509,hsa_circRNA_101473,hsa_circRNA_005931,hsa_circRNA_100674,hsa_circRNA_067130,hsa_circRNA_053294,hsa_circRNA_102924,hsa_circRNA_104721,hsa_circRNA_000324,hsa_circRNA_102949,hsa_circRNA_000735,hsa_circRNA_000676,hsa_circRNA_101693,hsa_circRNA_027446,hsa_circRNA_103038,hsa_circRNA_000791,hsa_circRNA_006766,hsa_circRNA_105035,hsa_circRNA_405792,hsa_circRNA_405504,hsa_circRNA_005272,hsa_circRNA_400097,hsa_circRNA_063227,hsa_circRNA_004520,hsa_circRNA_103547,hsa_circRNA_100375,hsa_circRNA_404617,hsa_circRNA_028595,hsa_circRNA_091244,hsa_circRNA_017648,hsa_circRNA_034435,hsa_circRNA_085918,hsa_circRNA_103461,hsa_circRNA_001038,hsa_circRNA_004895,hsa_circRNA_004797,hsa_circRNA_092520,hsa_circRNA_011164,hsa_circRNA_000525,hsa_circRNA_405813,hsa_circRNA_000960,hsa_circRNA_103399,hsa_circRNA_406584,hsa_circRNA_026134,hsa_circRNA_051799,hsa_circRNA_402893,hsa_circRNA_001257,hsa_circRNA_000410,hsa_circRNA_102579,hsa_circRNA_403922,hsa_circRNA_104572,hsa_circRNA_103296,hsa_circRNA_063226,hsa_circRNA_102217,hsa_circRNA_090364,hsa_circRNA_102339,hsa_circRNA_000361,hsa_circRNA_000446,hsa_circRNA_000911,hsa_circRNA_405308,hsa_circRNA_102715,hsa_circRNA_405007,hsa_circRNA_403882,hsa_circRNA_102448,hsa_circRNA_405508,hsa_circRNA_100751,hsa_circRNA_001594,hsa_circRNA_104970,hsa_circRNA_405558,hsa_circRNA_091774,hsa_circRNA_102887,hsa_circRNA_100891,hsa_circRNA_101795,hsa_circRNA_043415,hsa_circRNA_404853,hsa_circRNA_402931,hsa_circRNA_402952,hsa_circRNA_032154,hsa_circRNA_405016,hsa_circRNA_104950,hsa_circRNA_007237,hsa_circRNA_016229,hsa_circRNA_001175,hsa_circRNA_402505,hsa_circRNA_088030,hsa_circRNA_103403,hsa_circRNA_104850,hsa_circRNA_104851,hsa_circRNA_101061,hsa_circRNA_100497,hsa_circRNA_049382,hsa_circRNA_022429,hsa_circRNA_100258,hsa_circRNA_405324,hsa_circRNA_405634,hsa_circRNA_101707,hsa_circRNA_102417,hsa_circRNA_008115,hsa_circRNA_400015,hsa_circRNA_104951,hsa_circRNA_055855,hsa_circRNA_007586,hsa_circRNA_045361,hsa_circRNA_100750,hsa_circRNA_100236,hsa_circRNA_407344,hsa_circRNA_037128,hsa_circRNA_100615,hsa_circRNA_045920,hsa_circRNA_001920,hsa_circRNA_051933,hsa_circRNA_007418,hsa_circRNA_007215,hsa_circRNA_104109,hsa_circRNA_024246,hsa_circRNA_103551,hsa_circRNA_103164,hsa_circRNA_001000,hsa_circRNA_092393,hsa_circRNA_102575,hsa_circRNA_001225,hsa_circRNA_004045,hsa_circRNA_405264,hsa_circRNA_092388,hsa_circRNA_000865,hsa_circRNA_102328,hsa_circRNA_101592,hsa_circRNA_104205,hsa_circRNA_055440,hsa_circRNA_000694,hsa_circRNA_100178,hsa_circRNA_102573,hsa_circRNA_001357,hsa_circRNA_027234,hsa_circRNA_405962,hsa_circRNA_401091,hsa_circRNA_027023,hsa_circRNA_092378,hsa_circRNA_102774,hsa_circRNA_090920,hsa_circRNA_014213,hsa_circRNA_101144,hsa_circRNA_017693,hsa_circRNA_066331,hsa_circRNA_102062,hsa_circRNA_039830,hsa_circRNA_001029,hsa_circRNA_104103,hsa_circRNA_406715,hsa_circRNA_001526,hsa_circRNA_004041,hsa_circRNA_000113,hsa_circRNA_100570,hsa_circRNA_100505,hsa_circRNA_001914,hsa_circRNA_000404,hsa_circRNA_000403,hsa_circRNA_400031,hsa_circRNA_104506,hsa_circRNA_089414,hsa_circRNA_060056,hsa_circRNA_020748,hsa_circRNA_407292,hsa_circRNA_000692,hsa_circRNA_071981,hsa_circRNA_001298,hsa_circRNA_103400,hsa_circRNA_003201,hsa_circRNA_007700,hsa_circRNA_103402,hsa_circRNA_000103,hsa_circRNA_103700,hsa_circRNA_006002,hsa_circRNA_104756,hsa_circRNA_042079,hsa_circRNA_004594,hsa_circRNA_007273,hsa_circRNA_406832,hsa_circRNA_001589,hsa_circRNA_101644,hsa_circRNA_103290,hsa_circRNA_049241,hsa_circRNA_007081,hsa_circRNA_405325,hsa_circRNA_063179,hsa_circRNA_060539,hsa_circRNA_104341,hsa_circRNA_052282,hsa_circRNA_101924,hsa_circRNA_017695,hsa_circRNA_102447,hsa_circRNA_029301,hsa_circRNA_089386,hsa_circRNA_101323,hsa_circRNA_102583,hsa_circRNA_050963,hsa_circRNA_400540,hsa_circRNA_406997,hsa_circRNA_085931,hsa_circRNA_401746,hsa_circRNA_102441,hsa_circRNA_043552,hsa_circRNA_017077,hsa_circRNA_101571,hsa_circRNA_048963,hsa_circRNA_000816,hsa_circRNA_038632,hsa_circRNA_000809,hsa_circRNA_100503,hsa_circRNA_000342,hsa_circRNA_405619,hsa_circRNA_103278,hsa_circRNA_406326,hsa_circRNA_101937,hsa_circRNA_405798,hsa_circRNA_061900,hsa_circRNA_102555,hsa_circRNA_049377,hsa_circRNA_007884,hsa_circRNA_102483,hsa_circRNA_101798,hsa_circRNA_001369,hsa_circRNA_006153,hsa_circRNA_100569,hsa_circRNA_010210,hsa_circRNA_030885,hsa_circRNA_104110,hsa_circRNA_400032 | hsa_circRNA_033392,hsa_circRNA_000799,hsa_circRNA_103303,hsa_circRNA_033388,hsa_circRNA_104175,hsa_circRNA_102673,hsa_circRNA_102674,hsa_circRNA_008208,hsa_circRNA_403127,hsa_circRNA_407193,hsa_circRNA_100373,hsa_circRNA_104502,hsa_circRNA_405759,hsa_circRNA_104212,hsa_circRNA_102676,hsa_circRNA_104210,hsa_circRNA_104419,hsa_circRNA_401957,hsa_circRNA_100257,hsa_circRNA_003632,hsa_circRNA_104418,hsa_circRNA_103364,hsa_circRNA_008901,hsa_circRNA_102829,hsa_circRNA_103886,hsa_circRNA_008534,hsa_circRNA_404337,hsa_circRNA_000156,hsa_circRNA_101028,hsa_circRNA_104174,hsa_circRNA_001642,hsa_circRNA_400935,hsa_circRNA_005671,hsa_circRNA_402954,hsa_circRNA_100620,hsa_circRNA_002115,hsa_circRNA_400659,hsa_circRNA_057948,hsa_circRNA_012964,hsa_circRNA_402272,hsa_circRNA_102761,hsa_circRNA_102103,hsa_circRNA_100230,hsa_circRNA_087264,hsa_circRNA_103737,hsa_circRNA_103360,hsa_circRNA_402355,hsa_circRNA_001698,hsa_circRNA_031959,hsa_circRNA_103823,hsa_circRNA_104207,hsa_circRNA_101434,hsa_circRNA_404987,hsa_circRNA_103363,hsa_circRNA_103365,hsa_circRNA_003154,hsa_circRNA_103397,hsa_circRNA_004669,hsa_circRNA_103634,hsa_circRNA_100126,hsa_circRNA_104792,hsa_circRNA_400277,hsa_circRNA_402961,hsa_circRNA_074270,hsa_circRNA_104727,hsa_circRNA_101041,hsa_circRNA_003942,hsa_circRNA_055947,hsa_circRNA_030239,hsa_circRNA_056204,hsa_circRNA_100683,hsa_circRNA_102285,hsa_circRNA_103467,hsa_circRNA_103522,hsa_circRNA_102797,hsa_circRNA_025696,hsa_circRNA_100229,hsa_circRNA_081028,hsa_circRNA_405566,hsa_circRNA_104214,hsa_circRNA_102665,hsa_circRNA_406545,hsa_circRNA_028671,hsa_circRNA_101172,hsa_circRNA_005310,hsa_circRNA_102902,hsa_circRNA_091365,hsa_circRNA_102758,hsa_circRNA_405772,hsa_circRNA_002387,hsa_circRNA_001531,hsa_circRNA_104173,hsa_circRNA_002603,hsa_circRNA_102696,hsa_circRNA_404780,hsa_circRNA_100463,hsa_circRNA_104761,hsa_circRNA_102311,hsa_circRNA_400285,hsa_circRNA_004800,hsa_circRNA_100702,hsa_circRNA_405944,hsa_circRNA_406975,hsa_circRNA_008757,hsa_circRNA_104167,hsa_circRNA_002682,hsa_circRNA_013190,hsa_circRNA_002919,hsa_circRNA_103398,hsa_circRNA_104161,hsa_circRNA_100228,hsa_circRNA_100700,hsa_circRNA_406724,hsa_circRNA_072917,hsa_circRNA_001862,hsa_circRNA_403985,hsa_circRNA_104155,hsa_circRNA_092450,hsa_circRNA_405237,hsa_circRNA_066814,hsa_circRNA_104127,hsa_circRNA_103691,hsa_circRNA_101595,hsa_circRNA_002210,hsa_circRNA_073848,hsa_circRNA_100907,hsa_circRNA_057950,hsa_circRNA_406146,hsa_circRNA_400889,hsa_circRNA_103989,hsa_circRNA_100418,hsa_circRNA_050545,hsa_circRNA_102848,hsa_circRNA_404941,hsa_circRNA_078175,hsa_circRNA_016040,hsa_circRNA_105045,hsa_circRNA_104655,hsa_circRNA_103884,hsa_circRNA_104580,hsa_circRNA_401210,hsa_circRNA_406465,hsa_circRNA_406557,hsa_circRNA_039195,hsa_circRNA_102146,hsa_circRNA_001473,hsa_circRNA_401355,hsa_circRNA_103473,hsa_circRNA_077627,hsa_circRNA_077628,hsa_circRNA_002237,hsa_circRNA_055243,hsa_circRNA_103817,hsa_circRNA_009541,hsa_circRNA_101586,hsa_circRNA_007563,hsa_circRNA_015374,hsa_circRNA_103822,hsa_circRNA_103133,hsa_circRNA_101827,hsa_circRNA_406817,hsa_circRNA_103565,hsa_circRNA_102030,hsa_circRNA_101848,hsa_circRNA_018380,hsa_circRNA_006482,hsa_circRNA_102105,hsa_circRNA_002781,hsa_circRNA_008974,hsa_circRNA_402340,hsa_circRNA_092585,hsa_circRNA_103641,hsa_circRNA_101175,hsa_circRNA_092561,hsa_circRNA_406493,hsa_circRNA_086306,hsa_circRNA_018405,hsa_circRNA_104213,hsa_circRNA_002223,hsa_circRNA_082778,hsa_circRNA_103366,hsa_circRNA_104528,hsa_circRNA_017037,hsa_circRNA_031830,hsa_circRNA_083061,hsa_circRNA_001535,hsa_circRNA_100401,hsa_circRNA_103625,hsa_circRNA_103432,hsa_circRNA_104211,hsa_circRNA_406446,hsa_circRNA_100227,hsa_circRNA_011222,hsa_circRNA_002292,hsa_circRNA_100628,hsa_circRNA_404980,hsa_circRNA_100701,hsa_circRNA_101030,hsa_circRNA_102879,hsa_circRNA_403063,hsa_circRNA_008332,hsa_circRNA_054326,hsa_circRNA_009068,hsa_circRNA_100138,hsa_circRNA_004407,hsa_circRNA_029998,hsa_circRNA_103991,hsa_circRNA_103076,hsa_circRNA_101022,hsa_circRNA_400717,hsa_circRNA_002254,hsa_circRNA_037911,hsa_circRNA_031831,hsa_circRNA_057727,hsa_circRNA_018834,hsa_circRNA_001475,hsa_circRNA_405331,hsa_circRNA_103109,hsa_circRNA_101044,hsa_circRNA_025554,hsa_circRNA_102502,hsa_circRNA_091025,hsa_circRNA_030069,hsa_circRNA_403829,hsa_circRNA_103821,hsa_circRNA_404925,hsa_circRNA_104192,hsa_circRNA_403817,hsa_circRNA_403314,hsa_circRNA_104641,hsa_circRNA_016039,hsa_circRNA_102718,hsa_circRNA_054963,hsa_circRNA_000078,hsa_circRNA_103362,hsa_circRNA_044839,hsa_circRNA_010811,hsa_circRNA_104236,hsa_circRNA_002045,hsa_circRNA_102855,hsa_circRNA_039400,hsa_circRNA_049792,hsa_circRNA_102757,hsa_circRNA_103790,hsa_circRNA_015808,hsa_circRNA_104424,hsa_circRNA_103755,hsa_circRNA_103356,hsa_circRNA_104765,hsa_circRNA_103631,hsa_circRNA_403680,hsa_circRNA_000433,hsa_circRNA_401118,hsa_circRNA_005372,hsa_circRNA_003558,hsa_circRNA_100653,hsa_circRNA_100452,hsa_circRNA_102652,hsa_circRNA_405594 |
