# Supplementary material for: Circular RNA regulatory network reveals cell–cell crosstalk in acute myeloid leukemia extramedullary infiltration
Source: J Transl Med. 2018 Dec 17;16:361. doi: 10.1186/s12967-018-1726-x (PMC6297994; doi:10.1186/s12967-018-1726-x)
Supplement: Supplementary file 2 — Additional file 2: Table S2. The list of dysregulated genes between EMI and non-EMI AML samples. [file 12967_2018_1726_MOESM2_ESM.docx]

| **Table S2 The list of dysregulated genes between EMI and non-EMI AML samples** | | | | | | |
| --- | --- | --- | --- | --- | --- | --- |
|  | **up-regulated genes** | **Fold change** |  |  | **Down-regulated genes** | **Fold change** |
| 1 | COL8A2 | 52.18186 |  | 1 | CT45A1 | 129.9291 |
| 2 | CES1 | 45.75388 |  | 2 | SPINK2 | 109.4294 |
| 3 | ACKR3 | 36.00811 |  | 3 | MYCN | 73.39003 |
| 4 | MPP3 | 34.42217 |  | 4 | LTBP1 | 64.69184 |
| 5 | LPIN3 | 34.24938 |  | 5 | C3orf80 | 59.75557 |
| 6 | VSIG4 | 31.65333 |  | 6 | CT45A5 | 57.53549 |
| 7 | NID1 | 27.27479 |  | 7 | COL4A5 | 44.50175 |
| 8 | UTS2 | 26.77208 |  | 8 | SAGE1 | 41.35703 |
| 9 | IL1R2 | 24.20453 |  | 9 | RASGRP3 | 34.96425 |
| 10 | OLFML2A | 23.742 |  | 10 | CLC | 34.61184 |
| 11 | LINC00937 | 22.58171 |  | 11 | SLC40A1 | 30.7822 |
| 12 | CDC42EP1 | 21.67664 |  | 12 | ABCA13 | 24.30081 |
| 13 | CYP27A1 | 20.0721 |  | 13 | TCN1 | 21.64418 |
| 14 | DAGLA | 19.18091 |  | 14 | DNAJC6 | 20.95107 |
| 15 | NAV1 | 18.37607 |  | 15 | lnc-KIAA0087-2 | 20.81422 |
| 16 | PRKCDBP | 17.94229 |  | 16 | APOC2 | 20.61864 |
| 17 | ASGR1 | 16.29026 |  | 17 | FAM81B | 19.48676 |
| 18 | ST14 | 16.10988 |  | 18 | CNRIP1 | 18.94162 |
| 19 | MYOF | 15.18352 |  | 19 | RHAG | 18.61636 |
| 20 | ZNF462 | 15.12432 |  | 20 | BMX | 17.8209 |
| 21 | PTGIR | 15.084 |  | 21 | ST6GALNAC1 | 17.80964 |
| 22 | C11orf96 | 13.12862 |  | 22 | CCDC3 | 17.73296 |
| 23 | HLA-DQB1 | 13.12241 |  | 23 | XK | 17.60915 |
| 24 | KLK1 | 13.01658 |  | 24 | SAMD12 | 17.54928 |
| 25 | CLEC4G | 12.49327 |  | 25 | SUCNR1 | 16.25589 |
| 26 | SLC8A1 | 11.82358 |  | 26 | RNASE3 | 12.58563 |
| 27 | TNFSF9 | 11.71711 |  | 27 | LUM | 11.8991 |
| 28 | GPR124 | 11.6253 |  | 28 | SEPP1 | 11.72563 |
| 29 | VENTX | 11.33687 |  | 29 | ANGPT1 | 11.56283 |
| 30 | EFCC1 | 11.21927 |  | 30 | PCAT18 | 11.47175 |
| 31 | ID1 | 11.1335 |  | 31 | PRG2 | 11.15979 |
| 32 | TIFAB | 10.92278 |  | 32 | RNASE2 | 11.08303 |
| 33 | EMILIN1 | 10.91761 |  | 33 | ADTRP | 10.82365 |
| 34 | SRGAP2B | 10.79268 |  | 34 | IFIT1B | 10.67353 |
| 35 | SLC37A2 | 10.57066 |  | 35 | MAP9 | 10.54964 |
| 36 | TMEM176A | 10.54753 |  | 36 | TUBB1 | 10.03663 |
| 37 | ADAMTSL4 | 10.41998 |  | 37 | CYP4F3 | 10.00192 |
| 38 | SLED1 | 10.36719 |  | 38 | LPAR4 | 9.688861 |
| 39 | SASH1 | 10.35678 |  | 39 | ZNF420 | 9.31951 |
| 40 | DLGAP3 | 10.04362 |  | 40 | HIST1H2AC | 9.264726 |
| 41 | SLAMF8 | 9.98258 |  | 41 | DPY19L2 | 9.202565 |
| 42 | FAM105A | 9.634399 |  | 42 | CA1 | 9.15608 |
| 43 | FGF13 | 9.580056 |  | 43 | VWF | 9.031111 |
| 44 | PAPSS2 | 9.52293 |  | 44 | PTH2R | 9.024278 |
| 45 | C1QA | 9.370119 |  | 45 | KLF5 | 8.440801 |
| 46 | KLHL30 | 9.320037 |  | 46 | INADL | 8.329891 |
| 47 | MLLT4 | 9.200288 |  | 47 | RHOXF2 | 8.292239 |
| 48 | ADCY9 | 9.091146 |  | 48 | ZNF100 | 8.007571 |
| 49 | SH3TC1 | 9.080181 |  | 49 | MSI2 | 7.780928 |
| 50 | GRASP | 8.989217 |  | 50 | FAM46C | 7.77462 |
| 51 | MTMR11 | 8.891027 |  | 51 | MGAT3 | 7.706554 |
| 52 | HLA-DRB1 | 8.669467 |  | 52 | SPTA1 | 7.664035 |
| 53 | PMP22 | 8.576494 |  | 53 | MYZAP | 7.423723 |
| 54 | HLA-DRB3 | 8.546825 |  | 54 | GUCY1B3 | 7.252793 |
| 55 | ZNF703 | 8.514749 |  | 55 | CRNDE | 7.241811 |
| 56 | HLA-DQB2 | 8.456147 |  | 56 | LCA5 | 7.222106 |
| 57 | VEGFA | 8.388498 |  | 57 | MMP7 | 7.200836 |
| 58 | HLA-DRB4 | 8.384288 |  | 58 | CLEC1B | 7.179577 |
| 59 | RNF175 | 8.379041 |  | 59 | OGN | 7.166739 |
| 60 | ATP10A | 8.214243 |  | 60 | HMGN5 | 7.163753 |
| 61 | PDK4 | 7.998038 |  | 61 | LOC400682 | 7.122762 |
| 62 | SSPO | 7.992518 |  | 62 | LOC441204 | 7.06208 |
| 63 | PLEKHG2 | 7.957609 |  | 63 | FECH | 6.913796 |
| 64 | SRGAP2 | 7.883669 |  | 64 | lnc-RP11-712L6.5.1-2 | 6.886918 |
| 65 | CD52 | 7.882275 |  | 65 | ACSM3 | 6.836216 |
| 66 | NSUN7 | 7.789644 |  | 66 | C2orf88 | 6.802149 |
| 67 | PAPLN | 7.730611 |  | 67 | RPS6KA5 | 6.587527 |
| 68 | HLA-DRB5 | 7.724075 |  | 68 | AKR1C4 | 6.53614 |
| 69 | CD1D | 7.538193 |  | 69 | KIAA1586 | 6.462439 |
| 70 | CMTM8 | 7.462668 |  | 70 | FASLG | 6.408244 |
| 71 | TBC1D8 | 7.448605 |  | 71 | SORBS1 | 6.400545 |
| 72 | CCR2 | 7.393429 |  | 72 | LOC100233156 | 6.399432 |
| 73 | HLA-DRB6 | 7.379005 |  | 73 | CCDC176 | 6.376952 |
| 74 | HLA-DMA | 7.35899 |  | 74 | DENND5B | 6.35238 |
| 75 | SIAE | 7.347764 |  | 75 | MEX3B | 6.327264 |
| 76 | CTSV | 7.296349 |  | 76 | ZSCAN31 | 6.318503 |
| 77 | PLK2 | 7.281331 |  | 77 | LOC100507195 | 6.277737 |
| 78 | NRP1 | 7.205199 |  | 78 | PARD6B | 6.273464 |
| 79 | TMEM176B | 7.179099 |  | 79 | NT5C3A | 6.263494 |
| 80 | RASSF4 | 7.156513 |  | 80 | KLF12 | 6.257989 |
| 81 | CD74 | 7.109837 |  | 81 | LOC645195 | 6.136806 |
| 82 | PLEKHG3 | 7.05793 |  | 82 | LEF1 | 6.079115 |
| 83 | PKIB | 7.048849 |  | 83 | KIAA1217 | 6.057726 |
| 84 | FLNB | 6.913264 |  | 84 | MINPP1 | 6.002945 |
| 85 | CSF1R | 6.902435 |  | 85 | LOC646652 | 5.972004 |
| 86 | ADAMTSL2 | 6.84738 |  | 86 | LNX2 | 5.93286 |
| 87 | PRKCA | 6.802812 |  | 87 | LOC256880 | 5.908056 |
| 88 | GPER1 | 6.786426 |  | 88 | SOHLH2 | 5.885763 |
| 89 | LOC100128242 | 6.765664 |  | 89 | CD40LG | 5.884229 |
| 90 | FGD4 | 6.754037 |  | 90 | LXN | 5.875881 |
| 91 | PDE4A | 6.582187 |  | 91 | MYCNOS | 5.871871 |
| 92 | VAV2 | 6.523849 |  | 92 | XLOC_l2_013314 | 5.86008 |
| 93 | GAA | 6.498005 |  | 93 | DHRS11 | 5.804481 |
| 94 | GSTM5 | 6.469872 |  | 94 | C2orf15 | 5.699412 |
| 95 | RNF112 | 6.454426 |  | 95 | FAM19A2 | 5.688039 |
| 96 | CACNA2D3 | 6.383967 |  | 96 | HIST2H2AA4 | 5.681371 |
| 97 | HLA-DRA | 6.376198 |  | 97 | OR2B6 | 5.676677 |
| 98 | FOSB | 6.35513 |  | 98 | LOC389834 | 5.653452 |
| 99 | lnc-TM4SF4-2 | 6.331586 |  | 99 | GLCCI1 | 5.649715 |
| 100 | MT1E | 6.310166 |  | 100 | RHOBTB1 | 5.643993 |
| 101 | FAM89A | 6.303739 |  | 101 | SSX2IP | 5.63775 |
| 102 | LOC100129846 | 6.158097 |  | 102 | DYNLRB2 | 5.632042 |
| 103 | SPARC | 6.125576 |  | 103 | SPIN4 | 5.591194 |
| 104 | UNC5B | 6.074581 |  | 104 | ZNF493 | 5.564083 |
| 105 | ACVRL1 | 6.0559 |  | 105 | LOC344887 | 5.556957 |
| 106 | CMKLR1 | 6.021172 |  | 106 | KDELC1 | 5.555458 |
| 107 | NR4A2 | 5.984459 |  | 107 | FBN1 | 5.525744 |
| 108 | SIK1 | 5.956557 |  | 108 | SH3BGRL2 | 5.505817 |
| 109 | ICAM5 | 5.927668 |  | 109 | TARP | 5.488363 |
| 110 | PDE2A | 5.926607 |  | 110 | HIST1H2BK | 5.47692 |
| 111 | CYP2S1 | 5.854277 |  | 111 | PPP1R27 | 5.43093 |
| 112 | SOCS3 | 5.821105 |  | 112 | ZNF175 | 5.427909 |
| 113 | VDR | 5.811524 |  | 113 | MFAP3L | 5.423882 |
| 114 | STK32C | 5.789062 |  | 114 | DPY19L1P1 | 5.351381 |
| 115 | BCAT1 | 5.749493 |  | 115 | EPM2AIP1 | 5.312901 |
| 116 | TRPS1 | 5.733777 |  | 116 | YES1 | 5.2976 |
| 117 | LOC729732 | 5.671498 |  | 117 | ZNF680 | 5.295256 |
| 118 | TMEM51 | 5.607743 |  | 118 | MYRIP | 5.271084 |
| 119 | MARVELD1 | 5.579875 |  | 119 | ZNF790 | 5.248149 |
| 120 | GPR162 | 5.507714 |  | 120 | PGM2L1 | 5.236919 |
| 121 | TRPM2 | 5.432805 |  | 121 | SP4 | 5.224569 |
| 122 | EPB41L2 | 5.416438 |  | 122 | COL6A5 | 5.214206 |
| 123 | IQSEC2 | 5.338168 |  | 123 | DPY19L2P2 | 5.125393 |
| 124 | MFSD2A | 5.268378 |  | 124 | MAP7 | 5.116087 |
| 125 | PLD4 | 5.248206 |  | 125 | KLHL9 | 5.075923 |
| 126 | ASB2 | 5.215931 |  | 126 | HIST1H1C | 5.06814 |
| 127 | PLXNA1 | 5.191642 |  | 127 | LOC100130920 | 5.043939 |
| 128 | RHOB | 5.177836 |  | 128 | NEXN | 5.031607 |
| 129 | FAM134B | 5.16114 |  | 129 | GCGR | 5.022002 |
| 130 | APP | 5.154183 |  | 130 | EPGN | 5.002754 |
| 131 | DFNB31 | 5.150477 |  | 131 | ATP7A | 4.984728 |
| 132 | IL27RA | 5.122372 |  | 132 | MBLAC2 | 4.966462 |
| 133 | FGD5 | 5.118877 |  | 133 | TWISTNB | 4.945971 |
| 134 | ANKRD65 | 5.089684 |  | 134 | ZNF254 | 4.923092 |
| 135 | TMEM121 | 5.082763 |  | 135 | CTSF | 4.914041 |
| 136 | CDKN1A | 5.079385 |  | 136 | ZNF566 | 4.881703 |
| 137 | GAREML | 5.039663 |  | 137 | CDK14 | 4.862168 |
| 138 | CCDC151 | 5.033343 |  | 138 | PLS1 | 4.852231 |
| 139 | IL6 | 5.028299 |  | 139 | HRH4 | 4.840615 |
| 140 | SCIMP | 5.002065 |  | 140 | NELL2 | 4.831733 |
| 141 | MT1B | 4.999975 |  | 141 | PFN1P2 | 4.824412 |
| 142 | ACY3 | 4.903265 |  | 142 | NDFIP2 | 4.799004 |
| 143 | lnc-LONRF1-2 | 4.89911 |  | 143 | BBS10 | 4.780709 |
| 144 | PLK3 | 4.889496 |  | 144 | LYSMD3 | 4.766955 |
| 145 | TNFRSF21 | 4.888385 |  | 145 | SPDYE8P | 4.755713 |
| 146 | MTSS1 | 4.884617 |  | 146 | ATP8B4 | 4.738651 |
| 147 | GPBAR1 | 4.813594 |  | 147 | SH2D1B | 4.73383 |
| 148 | FCGRT | 4.811463 |  | 148 | BPGM | 4.715922 |
| 149 | ZNF469 | 4.794832 |  | 149 | TMEM237 | 4.693659 |
| 150 | C9orf47 | 4.772176 |  | 150 | ZNF658 | 4.649868 |
| 151 | SLC4A11 | 4.737436 |  | 151 | ZNF569 | 4.645069 |
| 152 | UAP1L1 | 4.724075 |  | 152 | LEPR | 4.615339 |
| 153 | ZFP36 | 4.710404 |  | 153 | FAM46A | 4.605755 |
| 154 | NLRP3 | 4.695425 |  | 154 | TMEM106B | 4.588247 |
| 155 | CD180 | 4.67717 |  | 155 | AFF2 | 4.577849 |
| 156 | FAM72D | 4.663598 |  | 156 | ZNF43 | 4.565935 |
| 157 | LOC254896 | 4.655827 |  | 157 | STXBP5 | 4.525285 |
| 158 | CDCP1 | 4.647366 |  | 158 | ADH4 | 4.519287 |
| 159 | TPPP3 | 4.634254 |  | 159 | ANKRD50 | 4.498524 |
| 160 | MREG | 4.630999 |  | 160 | ZNF273 | 4.497919 |
| 161 | PTPRO | 4.603673 |  | 161 | TTC27 | 4.488891 |
| 162 | CTSZ | 4.567062 |  | 162 | ZNF257 | 4.460367 |
| 163 | TNFSF12 | 4.548852 |  | 163 | SLC16A14 | 4.444656 |
| 164 | PIM3 | 4.548404 |  | 164 | PDE5A | 4.429932 |
| 165 | LOC142937 | 4.536155 |  | 165 | LCTL | 4.418975 |
| 166 | ECHDC3 | 4.528036 |  | 166 | CCDC169 | 4.405402 |
| 167 | PPAPDC3 | 4.501954 |  | 167 | OCLN | 4.397675 |
| 168 | RPS6KA4 | 4.477256 |  | 168 | ZNF614 | 4.378972 |
| 169 | PIF1 | 4.474647 |  | 169 | DYX1C1 | 4.375604 |
| 170 | GAS2L1 | 4.468204 |  | 170 | BMP6 | 4.345336 |
| 171 | CEP55 | 4.418433 |  | 171 | ZNF10 | 4.33308 |
| 172 | SAPCD2 | 4.415684 |  | 172 | ZNF571 | 4.301319 |
| 173 | TOB1 | 4.414595 |  | 173 | DYNC2LI1 | 4.282733 |
| 174 | MCAM | 4.393647 |  | 174 | PDIK1L | 4.264853 |
| 175 | VAMP5 | 4.391745 |  | 175 | EPHA4 | 4.259427 |
| 176 | MAN1C1 | 4.358609 |  | 176 | KPNA5 | 4.252554 |
| 177 | HMOX1 | 4.277117 |  | 177 | OSBPL10 | 4.25108 |
| 178 | C1QB | 4.243928 |  | 178 | SETMAR | 4.239447 |
| 179 | MT1L | 4.214069 |  | 179 | ASB15 | 4.23335 |
| 180 | TKTL2 | 4.211512 |  | 180 | CAPS2 | 4.226663 |
| 181 | GRAMD1B | 4.21094 |  | 181 | DTWD2 | 4.215137 |
| 182 | LMNB1 | 4.173915 |  | 182 | HIST1H2AE | 4.155469 |
| 183 | LOC399900 | 4.156877 |  | 183 | SAMD9 | 4.146503 |
| 184 | HAVCR2 | 4.15357 |  | 184 | ZNF91 | 4.116448 |
| 185 | MT2A | 4.138766 |  | 185 | BBS12 | 4.11637 |
| 186 | ST6GALNAC2 | 4.128984 |  | 186 | CDK6 | 4.110393 |
| 187 | AMFR | 4.126726 |  | 187 | SPDYE5 | 4.095046 |
| 188 | ITGAL | 4.11661 |  | 188 | B3GALNT1 | 4.085783 |
| 189 | SPATS2L | 4.083097 |  | 189 | RGS9BP | 4.077971 |
| 190 | TMEM255B | 4.083061 |  | 190 | RNF144A | 4.077707 |
| 191 | DUSP2 | 4.07647 |  | 191 | ISPD | 4.067061 |
| 192 | RIN1 | 4.074265 |  | 192 | ZFP82 | 4.066595 |
| 193 | DUSP5 | 4.073275 |  | 193 | GNPDA2 | 4.061011 |
| 194 | ABCA3 | 4.069492 |  | 194 | ZNF304 | 4.059333 |
| 195 | PVRL2 | 4.064082 |  | 195 | FZD7 | 4.027833 |
| 196 | IFITM3 | 4.05137 |  | 196 | RBM12B | 4.012532 |
| 197 | PRKACG | 4.041112 |  | 197 | ZNF492 | 4.003594 |
| 198 | PPARGC1B | 4.03302 |  | 198 | DNAH14 | 4.001324 |
| 199 | PFKFB2 | 4.013974 |  | 199 | JADE1 | 3.990975 |
| 200 | PSRC1 | 3.98689 |  | 200 | AIG1 | 3.987476 |
| 201 | GSTT2 | 3.982996 |  | 201 | KIFAP3 | 3.976198 |
| 202 | FAM114A1 | 3.964797 |  | 202 | NME7 | 3.966645 |
| 203 | DISC1 | 3.964103 |  | 203 | TMEM67 | 3.938876 |
| 204 | KLHDC7B | 3.956593 |  | 204 | MSRB3 | 3.920112 |
| 205 | TYMP | 3.929386 |  | 205 | HNRNPU-AS1 | 3.89874 |
| 206 | RRAS | 3.927403 |  | 206 | ZFP1 | 3.881091 |
| 207 | ADSSL1 | 3.909737 |  | 207 | RAB33A | 3.879978 |
| 208 | APOBR | 3.902858 |  | 208 | ZNF436 | 3.878682 |
| 209 | ACTB | 3.894654 |  | 209 | GATA1 | 3.868796 |
| 210 | STX1A | 3.875311 |  | 210 | HES2 | 3.855361 |
| 211 | FGD2 | 3.872446 |  | 211 | TMEM217 | 3.85439 |
| 212 | SSTR4 | 3.862232 |  | 212 | FCRL2 | 3.851533 |
| 213 | CSAD | 3.858377 |  | 213 | DKFZP434I0714 | 3.850789 |
| 214 | NAA38 | 3.847283 |  | 214 | STK26 | 3.847282 |
| 215 | COMMD9 | 3.842331 |  | 215 | FAM179B | 3.838417 |
| 216 | SRRM2 | 3.837222 |  | 216 | PDGFA | 3.838141 |
| 217 | MTHFR | 3.826972 |  | 217 | KBTBD7 | 3.837889 |
| 218 | SLIT1 | 3.821165 |  | 218 | ZNF547 | 3.837195 |
| 219 | RCN3 | 3.820623 |  | 219 | ROPN1L | 3.82804 |
| 220 | QKI | 3.816721 |  | 220 | IGLL1 | 3.817132 |
| 221 | LOC100131820 | 3.8061 |  | 221 | CD28 | 3.812215 |
| 222 | SPRY1 | 3.804203 |  | 222 | SNORA22 | 3.806626 |
| 223 | LOC400863 | 3.799831 |  | 223 | KIAA1147 | 3.806563 |
| 224 | TFEB | 3.765797 |  | 224 | ZNF208 | 3.75681 |
| 225 | IL6ST | 3.757883 |  | 225 | THAP5 | 3.756671 |
| 226 | REEP5 | 3.74959 |  | 226 | CDKN2AIP | 3.751227 |
| 227 | DNAJB5 | 3.741407 |  | 227 | ZNF92 | 3.741423 |
| 228 | OAS1 | 3.724397 |  | 228 | TNIK | 3.732378 |
| 229 | CLSTN1 | 3.723742 |  | 229 | GNLY | 3.722276 |
| 230 | PVRL4 | 3.722679 |  | 230 | KIF5C | 3.71831 |
| 231 | GFRA2 | 3.703968 |  | 231 | KIAA1958 | 3.709105 |
| 232 | SPINT1 | 3.701913 |  | 232 | C7orf25 | 3.704963 |
| 233 | ECEL1P2 | 3.700372 |  | 233 | MMP10 | 3.70409 |
| 234 | WFS1 | 3.68497 |  | 234 | PIP5K1B | 3.673814 |
| 235 | MILR1 | 3.683935 |  | 235 | PIWIL4 | 3.65975 |
| 236 | TMEM88 | 3.682071 |  | 236 | ST8SIA6 | 3.649746 |
| 237 | ABI3 | 3.67429 |  | 237 | NPAT | 3.645377 |
| 238 | KIAA1211L | 3.669077 |  | 238 | ZNF550 | 3.643754 |
| 239 | S100A16 | 3.665086 |  | 239 | ZNF507 | 3.633016 |
| 240 | JUNB | 3.654494 |  | 240 | CCDC113 | 3.626049 |
| 241 | GM2A | 3.646929 |  | 241 | ANK3 | 3.583257 |
| 242 | CEBPD | 3.631431 |  | 242 | CASZ1 | 3.581487 |
| 243 | SSH2 | 3.631372 |  | 243 | PELO | 3.577118 |
| 244 | HLA-DPB2 | 3.62505 |  | 244 | OMD | 3.559767 |
| 245 | OAS2 | 3.624505 |  | 245 | TEX30 | 3.550739 |
| 246 | PRR27 | 3.611033 |  | 246 | SKP1 | 3.532788 |
| 247 | ADAMTSL3 | 3.608325 |  | 247 | GSKIP | 3.520854 |
| 248 | PER1 | 3.575018 |  | 248 | CAT | 3.512671 |
| 249 | CEP164 | 3.574071 |  | 249 | E2F5 | 3.497262 |
| 250 | C20orf27 | 3.568601 |  | 250 | WDR35 | 3.484597 |
| 251 | FAM57A | 3.559856 |  | 251 | PLA2G1B | 3.483455 |
| 252 | TSPAN4 | 3.557538 |  | 252 | ERAP1 | 3.480137 |
| 253 | RAB3IL1 | 3.544919 |  | 253 | TRIM23 | 3.477054 |
| 254 | LOC441268 | 3.540669 |  | 254 | ABHD17B | 3.477031 |
| 255 | UBXN11 | 3.5332 |  | 255 | FAM200A | 3.471516 |
| 256 | MOGAT1 | 3.523641 |  | 256 | TRIM24 | 3.468362 |
| 257 | MAP3K6 | 3.522958 |  | 257 | PPM1D | 3.457057 |
| 258 | NRXN3 | 3.518303 |  | 258 | CASP3 | 3.456406 |
| 259 | BAX | 3.51376 |  | 259 | L3MBTL1 | 3.454459 |
| 260 | TNFRSF10A | 3.488142 |  | 260 | ZNF222 | 3.451253 |
| 261 | OR8U1 | 3.481973 |  | 261 | LOC728975 | 3.44734 |
| 262 | GPRIN3 | 3.448941 |  | 262 | CEP70 | 3.445787 |
| 263 | NTSR1 | 3.444859 |  | 263 | ZBTB41 | 3.432368 |
| 264 | PLCB3 | 3.442555 |  | 264 | C12orf29 | 3.431537 |
| 265 | TCIRG1 | 3.432507 |  | 265 | UBE2E4P | 3.430362 |
| 266 | TSPAN14 | 3.427428 |  | 266 | IFT81 | 3.428458 |
| 267 | WTAP | 3.400929 |  | 267 | ABHD10 | 3.424015 |
| 268 | PAG1 | 3.399864 |  | 268 | CCDC62 | 3.4219 |
| 269 | ADAP1 | 3.396954 |  | 269 | ZNF501 | 3.40671 |
| 270 | CITED4 | 3.394542 |  | 270 | RLN2 | 3.404462 |
| 271 | MMP17 | 3.385447 |  | 271 | FGFBP3 | 3.395832 |
| 272 | F2RL1 | 3.381721 |  | 272 | METTL18 | 3.391613 |
| 273 | FSTL3 | 3.369506 |  | 273 | ZNF627 | 3.384251 |
| 274 | SH2B2 | 3.335112 |  | 274 | RAB2B | 3.378509 |
| 275 | CLEC3B | 3.329211 |  | 275 | ZNF557 | 3.375473 |
| 276 | P2RX7 | 3.329094 |  | 276 | DNAJB9 | 3.371809 |
| 277 | RELL2 | 3.317918 |  | 277 | NAPEPLD | 3.36731 |
| 278 | TBL1X | 3.316359 |  | 278 | CRY1 | 3.364406 |
| 279 | MYRF | 3.314733 |  | 279 | KRIT1 | 3.363007 |
| 280 | ASIC4 | 3.307803 |  | 280 | lnc-PDZD8-1 | 3.352606 |
| 281 | HCRTR1 | 3.305822 |  | 281 | C12orf73 | 3.350707 |
| 282 | LOC100128494 | 3.301631 |  | 282 | MYLK4 | 3.350098 |
| 283 | ZFAT | 3.300458 |  | 283 | PHF14 | 3.345464 |
| 284 | CCDC50 | 3.299575 |  | 284 | LOC728903 | 3.34426 |
| 285 | EFEMP2 | 3.299251 |  | 285 | HSF5 | 3.344103 |
| 286 | GLIS3 | 3.296856 |  | 286 | METTL15 | 3.340664 |
| 287 | RRP12 | 3.281398 |  | 287 | CEP170 | 3.335796 |
| 288 | MGAT1 | 3.277778 |  | 288 | YAE1D1 | 3.33472 |
| 289 | MICAL1 | 3.276458 |  | 289 | C12orf66 | 3.314519 |
| 290 | MAD1L1 | 3.272912 |  | 290 | lnc-SDR42E1-1 | 3.313394 |
| 291 | BCL3 | 3.259512 |  | 291 | ZNF624 | 3.31319 |
| 292 | SSC4D | 3.25942 |  | 292 | TSPYL4 | 3.307593 |
| 293 | DMD | 3.255291 |  | 293 | MIRLET7BHG | 3.307049 |
| 294 | KIAA0930 | 3.251664 |  | 294 | ZNF181 | 3.299001 |
| 295 | APOL3 | 3.250728 |  | 295 | GDF11 | 3.298033 |
| 296 | OGFR | 3.240145 |  | 296 | MSANTD4 | 3.294601 |
| 297 | CALHM2 | 3.230081 |  | 297 | LCMT2 | 3.292042 |
| 298 | C19orf38 | 3.227876 |  | 298 | USP53 | 3.285273 |
| 299 | ZNF575 | 3.225906 |  | 299 | MIB1 | 3.279517 |
| 300 | TRPV2 | 3.224107 |  | 300 | RNF219 | 3.269894 |
| 301 | MGC12916 | 3.217869 |  | 301 | ZBTB26 | 3.269882 |
| 302 | IZUMO1R | 3.209271 |  | 302 | ANXA2R | 3.267023 |
| 303 | EIF4EBP1 | 3.199182 |  | 303 | TP53RK | 3.255475 |
| 304 | ARHGEF10 | 3.198365 |  | 304 | HBP1 | 3.255034 |
| 305 | TRIM36 | 3.198319 |  | 305 | LZTFL1 | 3.252468 |
| 306 | RARA | 3.194986 |  | 306 | ZEB1-AS1 | 3.252407 |
| 307 | CSRNP1 | 3.193824 |  | 307 | HIST1H1T | 3.251495 |
| 308 | GALK1 | 3.185953 |  | 308 | FBXO36 | 3.250109 |
| 309 | NUPR1 | 3.181047 |  | 309 | PHOSPHO2 | 3.249873 |
| 310 | CNN2 | 3.167636 |  | 310 | ZNF644 | 3.239135 |
| 311 | ASS1 | 3.163624 |  | 311 | EPDR1 | 3.232565 |
| 312 | TRIM14 | 3.15549 |  | 312 | TMEM64 | 3.210001 |
| 313 | LRRC37A2 | 3.150518 |  | 313 | LOC100130093 | 3.203778 |
| 314 | C19orf84 | 3.144778 |  | 314 | RRP15 | 3.202444 |
| 315 | ATXN2L | 3.118787 |  | 315 | CCDC121 | 3.202256 |
| 316 | TTC7A | 3.117908 |  | 316 | TRIM68 | 3.201591 |
| 317 | HLA-F | 3.115968 |  | 317 | ANO2 | 3.19956 |
| 318 | SPACA6P | 3.107352 |  | 318 | TMEM60 | 3.188512 |
| 319 | OPLAH | 3.102213 |  | 319 | TRMT10C | 3.185032 |
| 320 | GJD3 | 3.09065 |  | 320 | PEX12 | 3.183877 |
| 321 | CYTIP | 3.082215 |  | 321 | NUCB2 | 3.179699 |
| 322 | PLXNB2 | 3.077579 |  | 322 | MPP5 | 3.174008 |
| 323 | TJP2 | 3.067887 |  | 323 | XLOC_l2_011265 | 3.173954 |
| 324 | GRK5 | 3.0671 |  | 324 | PIK3R1 | 3.169319 |
| 325 | HSD3B2 | 3.058491 |  | 325 | LOC283028 | 3.159867 |
| 326 | BAALC | 3.051383 |  | 326 | FBXO28 | 3.158938 |
| 327 | B3GNT3 | 3.051262 |  | 327 | TTC30B | 3.155371 |
| 328 | C9orf91 | 3.049832 |  | 328 | TTC9C | 3.1547 |
| 329 | SPATA13 | 3.049333 |  | 329 | STK17A | 3.13978 |
| 330 | ZNF821 | 3.044312 |  | 330 | CEP76 | 3.138838 |
| 331 | ALDH3B1 | 3.036795 |  | 331 | SNHG8 | 3.136986 |
| 332 | LCN15 | 3.022524 |  | 332 | ZNF654 | 3.130939 |
| 333 | ASTN2 | 3.012857 |  | 333 | SERF1A | 3.128441 |
| 334 | SLC13A2 | 3.009478 |  | 334 | STAM | 3.128097 |
| 335 | FUOM | 3.004346 |  | 335 | CTSW | 3.127855 |
| 336 | C14orf80 | 2.986021 |  | 336 | KBTBD6 | 3.124322 |
| 337 | TRIB1 | 2.980993 |  | 337 | DNM3 | 3.12349 |
| 338 | ATP1A1 | 2.979871 |  | 338 | lnc-WWP2-1 | 3.123013 |
| 339 | SHB | 2.976071 |  | 339 | KIAA1024 | 3.122468 |
| 340 | CEP85L | 2.972973 |  | 340 | FKBP7 | 3.121633 |
| 341 | SMCR6 | 2.972836 |  | 341 | PUS7L | 3.112957 |
| 342 | AGPAT6 | 2.971321 |  | 342 | BCKDHB | 3.104575 |
| 343 | SEPN1 | 2.965506 |  | 343 | TBX15 | 3.100216 |
| 344 | EHBP1L1 | 2.961632 |  | 344 | ZNF564 | 3.099365 |
| 345 | SYN1 | 2.958125 |  | 345 | SPG20-AS1 | 3.095146 |
| 346 | ETV1 | 2.95715 |  | 346 | FLJ46875 | 3.08979 |
| 347 | SLC12A9 | 2.950614 |  | 347 | C11orf1 | 3.088804 |
| 348 | FCHO1 | 2.948887 |  | 348 | FBXO3 | 3.0881 |
| 349 | GIMAP1 | 2.948652 |  | 349 | TYW5 | 3.087947 |
| 350 | TRIM34 | 2.934452 |  | 350 | LEKR1 | 3.086465 |
| 351 | TMEM54 | 2.929816 |  | 351 | RFX7 | 3.080747 |
| 352 | SLC31A1 | 2.928496 |  | 352 | ZNF35 | 3.076917 |
| 353 | TBL1Y | 2.923039 |  | 353 | MORC3 | 3.06991 |
| 354 | FERD3L | 2.921988 |  | 354 | FBXL20 | 3.065647 |
| 355 | TRIM11 | 2.914703 |  | 355 | TRAPPC2 | 3.058281 |
| 356 | ADCK1 | 2.90374 |  | 356 | SGOL2 | 3.057918 |
| 357 | TAPBP | 2.899021 |  | 357 | CENPJ | 3.057857 |
| 358 | GADD45B | 2.897267 |  | 358 | ZNF17 | 3.052031 |
| 359 | CD40 | 2.892979 |  | 359 | ABHD13 | 3.049987 |
| 360 | RAB3A | 2.892794 |  | 360 | PAXIP1-AS1 | 3.048004 |
| 361 | RPH3AL | 2.885866 |  | 361 | ANAPC10 | 3.04373 |
| 362 | ABCD1 | 2.885675 |  | 362 | FAHD1 | 3.043204 |
| 363 | PPP4R1L | 2.884642 |  | 363 | SNRPE | 3.036382 |
| 364 | TMEM108 | 2.880845 |  | 364 | PAQR3 | 3.030882 |
| 365 | IFITM2 | 2.874923 |  | 365 | FAM60A | 3.02662 |
| 366 | SNX27 | 2.868821 |  | 366 | PKIG | 3.009817 |
| 367 | PLEKHM1 | 2.864571 |  | 367 | AKR1C1 | 3.007001 |
| 368 | SLC27A1 | 2.860219 |  | 368 | SPG20 | 3.001152 |
| 369 | EPN2 | 2.858746 |  | 369 | NLRC3 | 2.994814 |
| 370 | CARD9 | 2.857969 |  | 370 | GFI1B | 2.99453 |
| 371 | CSRNP3 | 2.851455 |  | 371 | DYNLT3 | 2.993352 |
| 372 | AP2A2 | 2.842253 |  | 372 | SUGT1P3 | 2.993053 |
| 373 | SORBS3 | 2.838805 |  | 373 | OXNAD1 | 2.993024 |
| 374 | FAM171B | 2.838443 |  | 374 | SPATA5 | 2.991514 |
| 375 | TULP4 | 2.831335 |  | 375 | PRKCQ-AS1 | 2.9824 |
| 376 | ASCL2 | 2.830948 |  | 376 | KLHL20 | 2.974891 |
| 377 | SLC25A25 | 2.83074 |  | 377 | CCR9 | 2.973719 |
| 378 | TNFRSF10B | 2.819693 |  | 378 | CSTF1 | 2.973388 |
| 379 | PREX1 | 2.815646 |  | 379 | PGBD1 | 2.971925 |
| 380 | lnc-CAST-4 | 2.81527 |  | 380 | SMYD3 | 2.967366 |
| 381 | SMC1A | 2.814878 |  | 381 | METTL21A | 2.962364 |
| 382 | EPN1 | 2.806242 |  | 382 | KDM5B | 2.95751 |
| 383 | RAD23B | 2.800442 |  | 383 | AQP3 | 2.946263 |
| 384 | ARHGAP27 | 2.799297 |  | 384 | CROT | 2.9439 |
| 385 | IDH3A | 2.796717 |  | 385 | ZNF776 | 2.943264 |
| 386 | RHOBTB2 | 2.766429 |  | 386 | lnc-ANKRD11-5 | 2.942894 |
| 387 | IRF5 | 2.765032 |  | 387 | LRBA | 2.933528 |
| 388 | PARP10 | 2.759615 |  | 388 | IL15RA | 2.928988 |
| 389 | SLCO1A2 | 2.755576 |  | 389 | CEP97 | 2.927716 |
| 390 | LOC100129473 | 2.755217 |  | 390 | PCF11 | 2.924791 |
| 391 | AP1B1 | 2.751522 |  | 391 | RFC3 | 2.924501 |
| 392 | SCARB1 | 2.751431 |  | 392 | AGPAT5 | 2.922418 |
| 393 | RFX1 | 2.746355 |  | 393 | TRAPPC2B | 2.918678 |
| 394 | PACSIN2 | 2.738501 |  | 394 | MFSD8 | 2.918654 |
| 395 | TBC1D14 | 2.736409 |  | 395 | SEZ6 | 2.914085 |
| 396 | NFKBIA | 2.736222 |  | 396 | SPDYE1 | 2.913273 |
| 397 | KIFC2 | 2.730905 |  | 397 | LOC100130463 | 2.911372 |
| 398 | KIAA0040 | 2.718026 |  | 398 | TCTN1 | 2.910989 |
| 399 | ANXA6 | 2.713161 |  | 399 | RALGPS2 | 2.909046 |
| 400 | LPCAT4 | 2.712261 |  | 400 | C14orf28 | 2.904813 |
| 401 | ITPK1 | 2.708686 |  | 401 | PPIL6 | 2.902902 |
| 402 | MINOS1 | 2.708024 |  | 402 | lnc-MTERFD3-1 | 2.891391 |
| 403 | INSL3 | 2.705061 |  | 403 | MFAP1 | 2.889997 |
| 404 | TBCD | 2.701334 |  | 404 | LOC400756 | 2.887612 |
| 405 | BTN1A1 | 2.701048 |  | 405 | NR1D2 | 2.887516 |
| 406 | SNORA28 | 2.694287 |  | 406 | CREBRF | 2.886836 |
| 407 | DOT1L | 2.67954 |  | 407 | PEX3 | 2.885926 |
| 408 | RHOV | 2.677429 |  | 408 | IQCK | 2.884639 |
| 409 | ME1 | 2.673364 |  | 409 | ZNF260 | 2.882767 |
| 410 | COLGALT1 | 2.673253 |  | 410 | BBS1 | 2.880585 |
| 411 | LAMA4 | 2.658342 |  | 411 | MPC2 | 2.873779 |
| 412 | PSTPIP1 | 2.657077 |  | 412 | ERCC4 | 2.863217 |
| 413 | VAV1 | 2.655548 |  | 413 | DIEXF | 2.860566 |
| 414 | SH3KBP1 | 2.652951 |  | 414 | NFE2 | 2.860565 |
| 415 | FSD2 | 2.642698 |  | 415 | ZNF23 | 2.855957 |
| 416 | SHC1 | 2.630583 |  | 416 | MMADHC | 2.855621 |
| 417 | PTK6 | 2.626376 |  | 417 | CD247 | 2.854576 |
| 418 | FOXO1 | 2.624561 |  | 418 | TFB2M | 2.852211 |
| 419 | WDR49 | 2.623586 |  | 419 | PDZD8 | 2.8466 |
| 420 | PYCARD | 2.618366 |  | 420 | MOB4 | 2.842652 |
| 421 | HAAO | 2.617452 |  | 421 | OSCP1 | 2.841499 |
| 422 | ZNF385A | 2.614901 |  | 422 | ZNF780A | 2.840333 |
| 423 | MAP2K3 | 2.612541 |  | 423 | RASGRP1 | 2.83901 |
| 424 | EPHA8 | 2.607279 |  | 424 | BCDIN3D | 2.838476 |
| 425 | DNM1P46 | 2.603054 |  | 425 | ZNF831 | 2.838178 |
| 426 | TCF7L2 | 2.602619 |  | 426 | SOCS5 | 2.837455 |
| 427 | TREX1 | 2.600398 |  | 427 | RUNDC1 | 2.835636 |
| 428 | CACNA2D1 | 2.597731 |  | 428 | EXTL2 | 2.834795 |
| 429 | CDK5RAP3 | 2.594605 |  | 429 | ATP5E | 2.828413 |
| 430 | IMPDH1 | 2.583404 |  | 430 | IMMP1L | 2.828286 |
| 431 | SCRIB | 2.582811 |  | 431 | AGGF1 | 2.824502 |
| 432 | HAND1 | 2.580383 |  | 432 | POFUT1 | 2.82403 |
| 433 | DBNDD2 | 2.571428 |  | 433 | CKAP2 | 2.819307 |
| 434 | SYNGR2 | 2.57122 |  | 434 | ZNF615 | 2.818944 |
| 435 | PAM | 2.569896 |  | 435 | FANCF | 2.818048 |
| 436 | USP32 | 2.548986 |  | 436 | lnc-CX3CR1-2 | 2.817658 |
| 437 | S100A13 | 2.546419 |  | 437 | ZNF577 | 2.815522 |
| 438 | COX1 | 2.543188 |  | 438 | C12orf60 | 2.813181 |
| 439 | CDC42EP2 | 2.540381 |  | 439 | TBC1D19 | 2.811518 |
| 440 | ARHGEF28 | 2.536457 |  | 440 | ZNF180 | 2.811404 |
| 441 | USP13 | 2.534018 |  | 441 | FAM188B | 2.810982 |
| 442 | NPHS2 | 2.533655 |  | 442 | KIF20B | 2.808536 |
| 443 | ADCK5 | 2.526285 |  | 443 | LINC01004 | 2.802924 |
| 444 | MYO5B | 2.526036 |  | 444 | DNAAF2 | 2.802803 |
| 445 | LOC100128563 | 2.523689 |  | 445 | BBS9 | 2.802694 |
| 446 | ZNF318 | 2.517529 |  | 446 | B4GALT6 | 2.802406 |
| 447 | SRPX | 2.515465 |  | 447 | CAPRIN2 | 2.8023 |
| 448 | TNFAIP8L2 | 2.514513 |  | 448 | ZNF280D | 2.801908 |
| 449 | BOP1 | 2.514325 |  | 449 | COL12A1 | 2.801746 |
| 450 | ALDH16A1 | 2.505711 |  | 450 | WDR33 | 2.793373 |
| 451 | NAAA | 2.505016 |  | 451 | RBBP9 | 2.791328 |
| 452 | EPHB2 | 2.501125 |  | 452 | WDR19 | 2.781578 |
| 453 | FOSL2 | 2.497665 |  | 453 | STAM2 | 2.774635 |
| 454 | BAALC-AS2 | 2.493239 |  | 454 | ZNF486 | 2.766242 |
| 455 | ARHGEF40 | 2.488828 |  | 455 | SMC4 | 2.75513 |
| 456 | DAAM2 | 2.487453 |  | 456 | ZNF567 | 2.754035 |
| 457 | ANP32D | 2.484923 |  | 457 | ZFP62 | 2.75304 |
| 458 | ZMAT3 | 2.482492 |  | 458 | HAUS6 | 2.742882 |
| 459 | OR2L8 | 2.481504 |  | 459 | IBA57 | 2.737113 |
| 460 | KRT42P | 2.478839 |  | 460 | ALDH5A1 | 2.732472 |
| 461 | SHMT2 | 2.478199 |  | 461 | LOC729291 | 2.73173 |
| 462 | OAF | 2.477484 |  | 462 | RPL21 | 2.730701 |
| 463 | DLST | 2.473294 |  | 463 | ZNF227 | 2.719203 |
| 464 | LRRK1 | 2.468561 |  | 464 | EFCAB7 | 2.714263 |
| 465 | BTBD19 | 2.462604 |  | 465 | SNHG10 | 2.712719 |
| 466 | IPW | 2.462092 |  | 466 | BCAP29 | 2.711609 |
| 467 | GBGT1 | 2.461977 |  | 467 | ZNF200 | 2.711123 |
| 468 | AGAP3 | 2.461211 |  | 468 | ADPRM | 2.710775 |
| 469 | PIK3CD | 2.452605 |  | 469 | MSANTD3 | 2.710029 |
| 470 | PIWIL2 | 2.449818 |  | 470 | OSGEPL1 | 2.707061 |
| 471 | CD4 | 2.445235 |  | 471 | CFAP36 | 2.705899 |
| 472 | ATAD3C | 2.439661 |  | 472 | MFAP3 | 2.704683 |
| 473 | TAS2R5 | 2.438981 |  | 473 | ANKRD17 | 2.702402 |
| 474 | C11orf24 | 2.438542 |  | 474 | ALG10 | 2.700472 |
| 475 | GIPC1 | 2.438361 |  | 475 | ING3 | 2.697813 |
| 476 | SCART1 | 2.43834 |  | 476 | TRIM33 | 2.697651 |
| 477 | CCL19 | 2.435099 |  | 477 | NET1 | 2.695541 |
| 478 | PARP14 | 2.43311 |  | 478 | RNASEH2C | 2.692068 |
| 479 | LILRA1 | 2.429842 |  | 479 | CREB3L4 | 2.69077 |
| 480 | ADAM22 | 2.429829 |  | 480 | EBPL | 2.690516 |
| 481 | C4orf48 | 2.426083 |  | 481 | UNC119B | 2.685745 |
| 482 | FUBP3 | 2.423615 |  | 482 | TAF9B | 2.683888 |
| 483 | FAM120A | 2.421792 |  | 483 | FGFR1OP2 | 2.683382 |
| 484 | A1BG-AS1 | 2.421454 |  | 484 | ZNF738 | 2.683146 |
| 485 | CTBP2 | 2.414337 |  | 485 | ZNF582 | 2.682182 |
| 486 | WDR81 | 2.413078 |  | 486 | TTC28-AS1 | 2.67843 |
| 487 | LINC01105 | 2.412854 |  | 487 | ZRANB1 | 2.677494 |
| 488 | PTTG1IP | 2.412547 |  | 488 | LOC729080 | 2.676774 |
| 489 | ZFYVE19 | 2.411965 |  | 489 | TMEM59 | 2.668083 |
| 490 | ZNF366 | 2.407299 |  | 490 | MRPL50 | 2.664709 |
| 491 | KIF26B | 2.406817 |  | 491 | RC3H2 | 2.66159 |
| 492 | CRELD2 | 2.404864 |  | 492 | MKL2 | 2.660269 |
| 493 | TMEM75 | 2.403206 |  | 493 | ARMCX5 | 2.657568 |
| 494 | EDNRA | 2.402598 |  | 494 | DSCC1 | 2.656778 |
| 495 | MYO5A | 2.402235 |  | 495 | ZC3H8 | 2.648105 |
| 496 | PTGES2 | 2.396343 |  | 496 | CDK17 | 2.647936 |
| 497 | CD248 | 2.395045 |  | 497 | GEMIN8P4 | 2.645084 |
| 498 | AP2S1 | 2.392526 |  | 498 | ZNF701 | 2.644975 |
| 499 | ANP32A | 2.386746 |  | 499 | ORC4 | 2.644811 |
| 500 | IFITM4P | 2.384383 |  | 500 | COPG2 | 2.630564 |
| 501 | ARMC2 | 2.383918 |  | 501 | BDH2 | 2.628728 |
| 502 | STXBP5L | 2.381767 |  | 502 | TBC1D32 | 2.625189 |
| 503 | GUCY1B2 | 2.38136 |  | 503 | ZNF268 | 2.621444 |
| 504 | AGPAT4 | 2.380313 |  | 504 | ZNF572 | 2.620987 |
| 505 | MUSTN1 | 2.37929 |  | 505 | ZNF134 | 2.62033 |
| 506 | PTGER4P2-CDK2AP2P2 | 2.377593 |  | 506 | STRADB | 2.618227 |
| 507 | LILRB4 | 2.377233 |  | 507 | EPG5 | 2.617529 |
| 508 | TMC8 | 2.376559 |  | 508 | LNP1 | 2.6171 |
| 509 | SKIV2L | 2.37573 |  | 509 | NCK1 | 2.609474 |
| 510 | ATP1A4 | 2.374527 |  | 510 | DCAF10 | 2.60925 |
| 511 | AKAP13 | 2.370782 |  | 511 | ANKMY2 | 2.605378 |
| 512 | STARD3 | 2.368112 |  | 512 | LYRM5 | 2.602096 |
| 513 | NFYC | 2.36297 |  | 513 | CCT6B | 2.600536 |
| 514 | SIGMAR1 | 2.362519 |  | 514 | RPL21P44 | 2.597999 |
| 515 | DENND1C | 2.360592 |  | 515 | PAIP1 | 2.596148 |
| 516 | LOC439933 | 2.358674 |  | 516 | ARID1B | 2.592721 |
| 517 | LOC100131792 | 2.357795 |  | 517 | C2orf44 | 2.592274 |
| 518 | C17orf82 | 2.354903 |  | 518 | PRSS23 | 2.591053 |
| 519 | HLA-A | 2.348378 |  | 519 | lnc-ZNF345-1 | 2.590657 |
| 520 | TMEM161A | 2.347907 |  | 520 | THUMPD3-AS1 | 2.589368 |
| 521 | MBD3 | 2.347874 |  | 521 | SMIM17 | 2.588535 |
| 522 | GPR35 | 2.342893 |  | 522 | RIOK2 | 2.581488 |
| 523 | SLC25A19 | 2.340353 |  | 523 | NANOG | 2.581218 |
| 524 | ULK2 | 2.33585 |  | 524 | DBT | 2.580842 |
| 525 | BMF | 2.334757 |  | 525 | CHIC2 | 2.574931 |
| 526 | STARD5 | 2.330764 |  | 526 | ITGA6 | 2.574682 |
| 527 | OR2L3 | 2.327994 |  | 527 | GBA3 | 2.572654 |
| 528 | ACOT11 | 2.327462 |  | 528 | MORN2 | 2.571987 |
| 529 | ZNF511 | 2.32568 |  | 529 | AMD1 | 2.571149 |
| 530 | ANO8 | 2.317275 |  | 530 | SPDYE3 | 2.570668 |
| 531 | APBB1IP | 2.317236 |  | 531 | ELMOD2 | 2.569154 |
| 532 | ASCC3 | 2.314002 |  | 532 | SERF1B | 2.56915 |
| 533 | CCDC153 | 2.31204 |  | 533 | CDC37L1 | 2.564592 |
| 534 | PMAIP1 | 2.310539 |  | 534 | LOC541467 | 2.564539 |
| 535 | CTRC | 2.309487 |  | 535 | C7orf60 | 2.558026 |
| 536 | AGPAT3 | 2.309347 |  | 536 | EIF1AD | 2.557833 |
| 537 | CLEC12B | 2.308331 |  | 537 | MED13 | 2.557739 |
| 538 | SH3BP2 | 2.30579 |  | 538 | AGL | 2.54979 |
| 539 | EIF4G1 | 2.297177 |  | 539 | IFIT3 | 2.549359 |
| 540 | lnc-BTF3L4-2 | 2.296661 |  | 540 | NFU1 | 2.547998 |
| 541 | PLCB2 | 2.293679 |  | 541 | DNAJC18 | 2.547517 |
| 542 | TNIP1 | 2.29319 |  | 542 | SRPK1 | 2.546244 |
| 543 | HDGFRP2 | 2.291833 |  | 543 | LMBR1 | 2.540799 |
| 544 | PPP1R9B | 2.290986 |  | 544 | SCARNA13 | 2.536497 |
| 545 | LYPD5 | 2.288926 |  | 545 | CBX3P2 | 2.532759 |
| 546 | SH3BP1 | 2.281316 |  | 546 | IP6K2 | 2.523684 |
| 547 | LOC729680 | 2.280979 |  | 547 | VCPIP1 | 2.522587 |
| 548 | JMJD7-PLA2G4B | 2.275278 |  | 548 | KLHDC2 | 2.521154 |
| 549 | RRBP1 | 2.274874 |  | 549 | CEP57L1 | 2.518572 |
| 550 | ADAMTSL4-AS1 | 2.270722 |  | 550 | NPHP3 | 2.515233 |
| 551 | MAPKAPK3 | 2.270706 |  | 551 | AP4S1 | 2.514574 |
| 552 | ASAP3 | 2.268768 |  | 552 | ZNF670 | 2.513943 |
| 553 | DLG4 | 2.265579 |  | 553 | CASP6 | 2.513874 |
| 554 | HSPA5 | 2.265534 |  | 554 | TAF9 | 2.508 |
| 555 | CCL27 | 2.265498 |  | 555 | SMG8 | 2.505863 |
| 556 | MYC | 2.262801 |  | 556 | CNIH1 | 2.497379 |
| 557 | lnc-FOXI2-1 | 2.262134 |  | 557 | TET1 | 2.492596 |
| 558 | PRPF4 | 2.261326 |  | 558 | lnc-GUSB-5 | 2.491128 |
| 559 | CMTM7 | 2.258125 |  | 559 | KLHDC1 | 2.479875 |
| 560 | RXFP4 | 2.25413 |  | 560 | BCL11B | 2.46935 |
| 561 | AGPAT2 | 2.254027 |  | 561 | ICOS | 2.463247 |
| 562 | PCDH10 | 2.253706 |  | 562 | ZNF277 | 2.456741 |
| 563 | DPP3 | 2.252258 |  | 563 | TRMT13 | 2.455617 |
| 564 | TUBG1 | 2.245836 |  | 564 | LOC90246 | 2.452675 |
| 565 | LOC649305 | 2.244574 |  | 565 | WARS2 | 2.451272 |
| 566 | SPTBN5 | 2.244393 |  | 566 | SLC12A2 | 2.449871 |
| 567 | ANP32C | 2.244227 |  | 567 | TGFBRAP1 | 2.449323 |
| 568 | SNX30 | 2.243992 |  | 568 | MSANTD3-TMEFF1 | 2.449186 |
| 569 | RAB24 | 2.241558 |  | 569 | DLEU2L | 2.446836 |
| 570 | INO80 | 2.239703 |  | 570 | SAV1 | 2.446275 |
| 571 | UTRN | 2.239444 |  | 571 | TRIM4 | 2.444479 |
| 572 | IRF3 | 2.238599 |  | 572 | DBR1 | 2.443072 |
| 573 | PIK3CD-AS1 | 2.237474 |  | 573 | CYP7B1 | 2.442269 |
| 574 | SEBOX | 2.236933 |  | 574 | RPL23AP64 | 2.439045 |
| 575 | LINC00544 | 2.236393 |  | 575 | FAM175A | 2.438511 |
| 576 | ZDHHC16 | 2.227131 |  | 576 | ZNF138 | 2.437983 |
| 577 | ALDH3A1 | 2.220965 |  | 577 | FBXO30 | 2.437905 |
| 578 | SLC2A5 | 2.219133 |  | 578 | FBXL17 | 2.436668 |
| 579 | CPA4 | 2.218151 |  | 579 | TLR3 | 2.432268 |
| 580 | ENTPD6 | 2.216324 |  | 580 | ZNF320 | 2.429493 |
| 581 | BAIAP2-AS1 | 2.216171 |  | 581 | SUPT3H | 2.424958 |
| 582 | ABHD8 | 2.212515 |  | 582 | MAPKAPK5-AS1 | 2.423267 |
| 583 | CA5BP1 | 2.210055 |  | 583 | DPCD | 2.421599 |
| 584 | COASY | 2.200659 |  | 584 | SCCPDH | 2.419233 |
| 585 | GCSAM | 2.191737 |  | 585 | BCL2 | 2.417323 |
| 586 | NAGPA | 2.188259 |  | 586 | RAB33B | 2.415935 |
| 587 | LOC100130057 | 2.185507 |  | 587 | OR1L3 | 2.41261 |
| 588 | LINC00202-2 | 2.180707 |  | 588 | LDHB | 2.410426 |
| 589 | PROM2 | 2.180279 |  | 589 | ANKRD49 | 2.409559 |
| 590 | PRKX | 2.179981 |  | 590 | TAF1A | 2.405834 |
| 591 | ATF5 | 2.178796 |  | 591 | PAOX | 2.402755 |
| 592 | DUX4 | 2.174143 |  | 592 | ZNF83 | 2.402741 |
| 593 | KRTAP1-5 | 2.172525 |  | 593 | LINC00938 | 2.402305 |
| 594 | ATG101 | 2.171832 |  | 594 | PDCD2L | 2.401083 |
| 595 | BEST2 | 2.165982 |  | 595 | PPIL3 | 2.400088 |
| 596 | STK10 | 2.163936 |  | 596 | ZNF22 | 2.398635 |
| 597 | LINC01152 | 2.16079 |  | 597 | lnc-A2M-1 | 2.397743 |
| 598 | DISP2 | 2.159496 |  | 598 | METAP2 | 2.397197 |
| 599 | FKRP | 2.159238 |  | 599 | CCP110 | 2.396701 |
| 600 | P2RX4 | 2.152248 |  | 600 | NOL11 | 2.39572 |
| 601 | EVPLL | 2.150684 |  | 601 | MYNN | 2.395682 |
| 602 | KRTAP5-6 | 2.14939 |  | 602 | ALG10B | 2.391727 |
| 603 | C7orf13 | 2.147101 |  | 603 | UBE2Q2P2 | 2.3905 |
| 604 | TOMM40L | 2.145042 |  | 604 | JRKL | 2.388746 |
| 605 | CAPN15 | 2.142995 |  | 605 | PIKFYVE | 2.387924 |
| 606 | WDR4 | 2.129652 |  | 606 | PTAR1 | 2.387335 |
| 607 | CORO7 | 2.123288 |  | 607 | GPAM | 2.387195 |
| 608 | PKDCC | 2.121181 |  | 608 | CP | 2.384145 |
| 609 | NUP214 | 2.1172 |  | 609 | C11orf58 | 2.383315 |
| 610 | CHMP4B | 2.111175 |  | 610 | PRR34-AS1 | 2.382472 |
| 611 | DPP9 | 2.109192 |  | 611 | C5orf28 | 2.38234 |
| 612 | SEMA3G | 2.107924 |  | 612 | MEX3C | 2.381862 |
| 613 | LINC00029 | 2.103757 |  | 613 | MEAF6 | 2.377255 |
| 614 | KSR1 | 2.103659 |  | 614 | SFT2D3 | 2.371584 |
| 615 | ZNF524 | 2.101216 |  | 615 | ZNF302 | 2.371527 |
| 616 | OAZ2 | 2.096308 |  | 616 | ACTR6 | 2.370788 |
| 617 | TRIM41 | 2.096141 |  | 617 | PNP | 2.369533 |
| 618 | SYDE1 | 2.09383 |  | 618 | LRRIQ3 | 2.367075 |
| 619 | VSIG2 | 2.091876 |  | 619 | LRRN3 | 2.366048 |
| 620 | KU-MEL-3 | 2.089217 |  | 620 | MS4A1 | 2.364178 |
| 621 | ALDH1L1 | 2.085653 |  | 621 | SRFBP1 | 2.364155 |
| 622 | FAM102B | 2.084217 |  | 622 | STAU2 | 2.364002 |
| 623 | MAP4 | 2.082791 |  | 623 | TWSG1 | 2.36209 |
| 624 | KCNH4 | 2.081545 |  | 624 | ZNF765 | 2.359463 |
| 625 | LRP1 | 2.076126 |  | 625 | PTCD2 | 2.35828 |
| 626 | CLCN6 | 2.073919 |  | 626 | COMMD6 | 2.356111 |
| 627 | NCLN | 2.073125 |  | 627 | WWTR1 | 2.354205 |
| 628 | GRIN2C | 2.071781 |  | 628 | TRUB1 | 2.352128 |
| 629 | RAD51D | 2.069182 |  | 629 | C10orf88 | 2.350086 |
| 630 | TVP23A | 2.068684 |  | 630 | SACS | 2.347711 |
| 631 | CSH2 | 2.066691 |  | 631 | FRS2 | 2.346426 |
| 632 | PROB1 | 2.059207 |  | 632 | CDADC1 | 2.344616 |
| 633 | CSF3R | 2.057947 |  | 633 | GPATCH2L | 2.332448 |
| 634 | lnc-ZNF479-2 | 2.057744 |  | 634 | lnc-PIGM-1 | 2.332249 |
| 635 | HYDIN | 2.056945 |  | 635 | LOC100128593 | 2.33158 |
| 636 | FAM160A2 | 2.05416 |  | 636 | ZSCAN32 | 2.330809 |
| 637 | CDK2AP2 | 2.053831 |  | 637 | TBC1D23 | 2.330547 |
| 638 | GNL1 | 2.048936 |  | 638 | TMTC3 | 2.32555 |
| 639 | PPP1R18 | 2.048142 |  | 639 | RSBN1L | 2.320271 |
| 640 | SLC26A6 | 2.047614 |  | 640 | ZNF770 | 2.320129 |
| 641 | UBE3B | 2.047043 |  | 641 | ST13 | 2.318825 |
| 642 | SRCIN1 | 2.043095 |  | 642 | EIF3J | 2.318625 |
| 643 | FAM74A1 | 2.040844 |  | 643 | ZFAND4 | 2.317159 |
| 644 | STK11IP | 2.040206 |  | 644 | RPS10 | 2.315705 |
| 645 | TCERG1L | 2.039482 |  | 645 | RRAGB | 2.315243 |
| 646 | MTMR14 | 2.035009 |  | 646 | RBM12 | 2.312435 |
| 647 | NDFIP1 | 2.034809 |  | 647 | PPP1R3D | 2.311648 |
| 648 | LOC143666 | 2.034435 |  | 648 | ZSCAN16-AS1 | 2.311456 |
| 649 | FKBP11 | 2.033766 |  | 649 | TRMT12 | 2.308737 |
| 650 | COG4 | 2.032691 |  | 650 | TRIM59 | 2.306044 |
| 651 | CLPS | 2.030816 |  | 651 | CETN2 | 2.30564 |
| 652 | LOC100129069 | 2.029032 |  | 652 | C12orf76 | 2.304521 |
| 653 | CXorf49B | 2.028498 |  | 653 | TSTD2 | 2.303393 |
| 654 | PPDPF | 2.027351 |  | 654 | G3BP2 | 2.301568 |
| 655 | TOR3A | 2.025693 |  | 655 | ZSCAN16 | 2.299967 |
| 656 | ESPN | 2.025288 |  | 656 | ANKRD7 | 2.296671 |
| 657 | OR51B5 | 2.023828 |  | 657 | DTWD1 | 2.296444 |
| 658 | ZNF32-AS3 | 2.014025 |  | 658 | GID8 | 2.295398 |
| 659 | CTDNEP1 | 2.011074 |  | 659 | FUBP1 | 2.2893 |
| 660 | FN3K | 2.009513 |  | 660 | HINT1 | 2.288477 |
| 661 | EFTUD2 | 2.007388 |  | 661 | TAX1BP1 | 2.288215 |
| 662 | SLIT3 | 2.005656 |  | 662 | C21orf91 | 2.286347 |
| 663 | EP400NL | 2.001364 |  | 663 | YEATS4 | 2.285292 |
|  |  |  |  | 664 | RAD1 | 2.285196 |
|  |  |  |  | 665 | NPB | 2.282658 |
|  |  |  |  | 666 | SMARCAD1 | 2.279447 |
|  |  |  |  | 667 | ZNF77 | 2.277144 |
|  |  |  |  | 668 | LOC646976 | 2.273805 |
|  |  |  |  | 669 | PLEKHA3 | 2.273088 |
|  |  |  |  | 670 | UHRF1BP1 | 2.271985 |
|  |  |  |  | 671 | ZSCAN26 | 2.271442 |
|  |  |  |  | 672 | RPS15A | 2.270946 |
|  |  |  |  | 673 | ABCE1 | 2.270664 |
|  |  |  |  | 674 | ZNF431 | 2.270456 |
|  |  |  |  | 675 | CNOT4 | 2.269757 |
|  |  |  |  | 676 | LOC102723552 | 2.266386 |
|  |  |  |  | 677 | PRMT9 | 2.264796 |
|  |  |  |  | 678 | TPGS2 | 2.263319 |
|  |  |  |  | 679 | SRP9 | 2.263045 |
|  |  |  |  | 680 | ZNF416 | 2.26291 |
|  |  |  |  | 681 | CDK12 | 2.260694 |
|  |  |  |  | 682 | KLRB1 | 2.260615 |
|  |  |  |  | 683 | lnc-B4GALT3-1 | 2.259677 |
|  |  |  |  | 684 | ACP1 | 2.257084 |
|  |  |  |  | 685 | ZRANB3 | 2.255868 |
|  |  |  |  | 686 | NDUFAF4P1 | 2.255112 |
|  |  |  |  | 687 | CCDC138 | 2.252318 |
|  |  |  |  | 688 | PTCD3 | 2.245845 |
|  |  |  |  | 689 | PRR3 | 2.243965 |
|  |  |  |  | 690 | NUP35 | 2.243473 |
|  |  |  |  | 691 | MAX | 2.243061 |
|  |  |  |  | 692 | GPLD1 | 2.240883 |
|  |  |  |  | 693 | RSBN1 | 2.240837 |
|  |  |  |  | 694 | CENPC | 2.24 |
|  |  |  |  | 695 | TMEM245 | 2.239807 |
|  |  |  |  | 696 | MPZL1 | 2.237743 |
|  |  |  |  | 697 | UBLCP1 | 2.237606 |
|  |  |  |  | 698 | MYOM1 | 2.236773 |
|  |  |  |  | 699 | CXorf56 | 2.236022 |
|  |  |  |  | 700 | PKN2 | 2.232016 |
|  |  |  |  | 701 | IFT74 | 2.231826 |
|  |  |  |  | 702 | FBXO8 | 2.23067 |
|  |  |  |  | 703 | POLR1D | 2.226668 |
|  |  |  |  | 704 | PRMT6 | 2.223989 |
|  |  |  |  | 705 | DUS4L | 2.222272 |
|  |  |  |  | 706 | UNC80 | 2.221357 |
|  |  |  |  | 707 | LOC641746 | 2.221097 |
|  |  |  |  | 708 | OR10R3P | 2.220899 |
|  |  |  |  | 709 | TDG | 2.220727 |
|  |  |  |  | 710 | SPIN2B | 2.219205 |
|  |  |  |  | 711 | KIAA1328 | 2.219187 |
|  |  |  |  | 712 | HLTF | 2.216281 |
|  |  |  |  | 713 | BLK | 2.215898 |
|  |  |  |  | 714 | APOLD1 | 2.214344 |
|  |  |  |  | 715 | DMRTC1 | 2.21397 |
|  |  |  |  | 716 | IMPDH2 | 2.212437 |
|  |  |  |  | 717 | GABPB1 | 2.21211 |
|  |  |  |  | 718 | TCEAL1 | 2.21204 |
|  |  |  |  | 719 | TCEAL5 | 2.211848 |
|  |  |  |  | 720 | MBNL1-AS1 | 2.211678 |
|  |  |  |  | 721 | AEBP2 | 2.210834 |
|  |  |  |  | 722 | PLD1 | 2.20864 |
|  |  |  |  | 723 | NFE2L2 | 2.206263 |
|  |  |  |  | 724 | TMTC4 | 2.205266 |
|  |  |  |  | 725 | CBX3 | 2.202953 |
|  |  |  |  | 726 | BTF3 | 2.201661 |
|  |  |  |  | 727 | PPP1R12A | 2.199868 |
|  |  |  |  | 728 | GTF2E1 | 2.193466 |
|  |  |  |  | 729 | DZANK1 | 2.184925 |
|  |  |  |  | 730 | FAM98A | 2.182707 |
|  |  |  |  | 731 | USP38 | 2.182588 |
|  |  |  |  | 732 | DCUN1D4 | 2.179451 |
|  |  |  |  | 733 | FNDC3A | 2.177627 |
|  |  |  |  | 734 | ZWILCH | 2.176315 |
|  |  |  |  | 735 | SUV39H2 | 2.171943 |
|  |  |  |  | 736 | ZNF398 | 2.168324 |
|  |  |  |  | 737 | PRDM2 | 2.167877 |
|  |  |  |  | 738 | SCGN | 2.164591 |
|  |  |  |  | 739 | CSTF2T | 2.163651 |
|  |  |  |  | 740 | G2E3 | 2.162531 |
|  |  |  |  | 741 | NDUFA4 | 2.15842 |
|  |  |  |  | 742 | ZXDB | 2.156447 |
|  |  |  |  | 743 | ZNF207 | 2.153408 |
|  |  |  |  | 744 | CCDC91 | 2.152193 |
|  |  |  |  | 745 | PIK3C3 | 2.151147 |
|  |  |  |  | 746 | NAA15 | 2.146355 |
|  |  |  |  | 747 | IGBP1 | 2.145369 |
|  |  |  |  | 748 | CARF | 2.144528 |
|  |  |  |  | 749 | PHC1 | 2.143241 |
|  |  |  |  | 750 | LCK | 2.14213 |
|  |  |  |  | 751 | ATG5 | 2.141481 |
|  |  |  |  | 752 | VSIG1 | 2.138558 |
|  |  |  |  | 753 | ZZZ3 | 2.136208 |
|  |  |  |  | 754 | USP37 | 2.135464 |
|  |  |  |  | 755 | SWT1 | 2.133158 |
|  |  |  |  | 756 | CD3D | 2.132323 |
|  |  |  |  | 757 | LOC100270804 | 2.130191 |
|  |  |  |  | 758 | LOC649294 | 2.129254 |
|  |  |  |  | 759 | FAM76B | 2.128865 |
|  |  |  |  | 760 | WASL | 2.128853 |
|  |  |  |  | 761 | PDCL | 2.127718 |
|  |  |  |  | 762 | RSL24D1 | 2.126646 |
|  |  |  |  | 763 | DNAJC12 | 2.125959 |
|  |  |  |  | 764 | PRDM16 | 2.124118 |
|  |  |  |  | 765 | C5orf42 | 2.121319 |
|  |  |  |  | 766 | HELQ | 2.120226 |
|  |  |  |  | 767 | ZNF33A | 2.118851 |
|  |  |  |  | 768 | IFT52 | 2.118767 |
|  |  |  |  | 769 | ARID2 | 2.118674 |
|  |  |  |  | 770 | RDH14 | 2.118359 |
|  |  |  |  | 771 | ZNF570 | 2.116417 |
|  |  |  |  | 772 | PAIP2B | 2.114787 |
|  |  |  |  | 773 | BRF2 | 2.113196 |
|  |  |  |  | 774 | ZNF326 | 2.107182 |
|  |  |  |  | 775 | CEP128 | 2.107083 |
|  |  |  |  | 776 | CAPN7 | 2.105158 |
|  |  |  |  | 777 | ZNF137P | 2.104681 |
|  |  |  |  | 778 | C3orf38 | 2.104425 |
|  |  |  |  | 779 | BLZF1 | 2.104161 |
|  |  |  |  | 780 | ARL2BP | 2.102184 |
|  |  |  |  | 781 | TADA1 | 2.101843 |
|  |  |  |  | 782 | GPR78 | 2.098661 |
|  |  |  |  | 783 | POLR1C | 2.097247 |
|  |  |  |  | 784 | FASTKD3 | 2.097237 |
|  |  |  |  | 785 | BTBD1 | 2.096745 |
|  |  |  |  | 786 | TMPRSS9 | 2.0958 |
|  |  |  |  | 787 | ZNF417 | 2.094927 |
|  |  |  |  | 788 | ZNF655 | 2.094574 |
|  |  |  |  | 789 | KDM3A | 2.092891 |
|  |  |  |  | 790 | EGLN3 | 2.092477 |
|  |  |  |  | 791 | RPL10A | 2.09012 |
|  |  |  |  | 792 | LOC84843 | 2.08916 |
|  |  |  |  | 793 | CYCSP52 | 2.08684 |
|  |  |  |  | 794 | SRSF6 | 2.085868 |
|  |  |  |  | 795 | TAF1D | 2.084762 |
|  |  |  |  | 796 | DLG2 | 2.084643 |
|  |  |  |  | 797 | REN | 2.082255 |
|  |  |  |  | 798 | C16orf72 | 2.082063 |
|  |  |  |  | 799 | ACTR3B | 2.079582 |
|  |  |  |  | 800 | MYL2 | 2.078282 |
|  |  |  |  | 801 | STAG2 | 2.075945 |
|  |  |  |  | 802 | TOP2B | 2.07375 |
|  |  |  |  | 803 | ERI1 | 2.071771 |
|  |  |  |  | 804 | ZNF552 | 2.070796 |
|  |  |  |  | 805 | EIF4E | 2.070585 |
|  |  |  |  | 806 | CRCP | 2.067355 |
|  |  |  |  | 807 | C1orf131 | 2.065724 |
|  |  |  |  | 808 | SLC25A13 | 2.065444 |
|  |  |  |  | 809 | HDAC8 | 2.064141 |
|  |  |  |  | 810 | EAPP | 2.060405 |
|  |  |  |  | 811 | ZNF471 | 2.059476 |
|  |  |  |  | 812 | LARP1B | 2.057203 |
|  |  |  |  | 813 | FOPNL | 2.053546 |
|  |  |  |  | 814 | MCM9 | 2.053242 |
|  |  |  |  | 815 | RBMXL1 | 2.049939 |
|  |  |  |  | 816 | BTF3P11 | 2.048755 |
|  |  |  |  | 817 | PRIM2 | 2.047195 |
|  |  |  |  | 818 | ZNF12 | 2.046291 |
|  |  |  |  | 819 | DPY19L4 | 2.045747 |
|  |  |  |  | 820 | RPSAP58 | 2.042996 |
|  |  |  |  | 821 | CAMK4 | 2.042072 |
|  |  |  |  | 822 | ZNF596 | 2.03676 |
|  |  |  |  | 823 | MBIP | 2.036415 |
|  |  |  |  | 824 | CSNK2A1 | 2.035872 |
|  |  |  |  | 825 | UBASH3A | 2.03261 |
|  |  |  |  | 826 | ARMC8 | 2.031079 |
|  |  |  |  | 827 | IFIH1 | 2.031027 |
|  |  |  |  | 828 | RPL15 | 2.023255 |
|  |  |  |  | 829 | BZW2 | 2.021721 |
|  |  |  |  | 830 | ITGAE | 2.020413 |
|  |  |  |  | 831 | ARMC10 | 2.014765 |
|  |  |  |  | 832 | SOCS4 | 2.014647 |
|  |  |  |  | 833 | OIP5-AS1 | 2.013589 |
|  |  |  |  | 834 | PPIP5K2 | 2.009873 |
|  |  |  |  | 835 | HGD | 2.009274 |
|  |  |  |  | 836 | GMPR2 | 2.006484 |
|  |  |  |  | 837 | LCLAT1 | 2.003192 |
|  |  |  |  | 838 | EIF4B | 2.000491 |
