# Supplementary material for: Circular RNA regulatory network reveals cell–cell crosstalk in acute myeloid leukemia extramedullary infiltration
Source: J Transl Med. 2018 Dec 17;16:361. doi: 10.1186/s12967-018-1726-x (PMC6297994; doi:10.1186/s12967-018-1726-x)
Supplement: Supplementary file 5 — Additional file 5: Table S5. KEGG pathway enrichment of upregulated genes between EMI and non-EMI AML samples. [file 12967_2018_1726_MOESM5_ESM.docx]

| **Table S5** KEGG pathway enrichment of upregulated genes between EMI and non-EMI AML samples | | | | |
| --- | --- | --- | --- | --- |
| Pathway ID | Definition | Fisher P value | Enrichment Score | Genes |
| hsa04015 | Rap1 signaling pathway - Homo sapiens (human) | 0.004204375 | 2.376299 | ACTB,ADCY9,APBB1IP,CSF1R,FGF13,ID1,ITGAL,MAP2K3,MLLT4,PIK3CD,PLCB2,PLCB3,PRKCA,RRAS,VAV2,VEGFA |
| hsa04612 | Antigen processing and presentation - Homo sapiens (human) | 5.72423E-07 | 6.242283 | CD4,CD74,HLA-A,HLA-DMA,HLA-DQB1,HLA-DRA,HLA-DRB1,HLA-DRB3,HLA-DRB4,HLA-DRB5,HLA-F,HSPA5,NFYC,TAPBP |
| hsa04621 | NOD-like receptor signaling pathway - Homo sapiens (human) | 0.003268762 | 2.485617 | CARD9,IL6,IRF3,NFKBIA,NLRP3,OAS1,OAS2,P2RX7,PLCB2,PLCB3,PSTPIP1,PYCARD,TRPM2,TRPV2 |
| hsa04640 | Hematopoietic cell lineage - Homo sapiens (human) | 4.61329E-05 | 4.33599 | CD1D,CD4,CSF1R,CSF3R,HLA-DMA,HLA-DQB1,HLA-DRA,HLA-DRB1,HLA-DRB3,HLA-DRB4,HLA-DRB5,IL1R2,IL6 |
| hsa04659 | Th17 cell differentiation - Homo sapiens (human) | 0.000129386 | 3.888113 | CD4,HLA-DMA,HLA-DQB1,HLA-DRA,HLA-DRB1,HLA-DRB3,HLA-DRB4,HLA-DRB5,IL27RA,IL6,IL6ST,NFKBIA,RARA |
| hsa04514 | Cell adhesion molecules (CAMs) - Homo sapiens (human) | 0.002257536 | 2.646365 | CD4,CD40,HLA-A,HLA-DMA,HLA-DQB1,HLA-DRA,HLA-DRB1,HLA-DRB3,HLA-DRB4,HLA-DRB5,HLA-F,ITGAL,NRXN3 |
| hsa04961 | Endocrine and other factor-regulated calcium reabsorption - Homo sapiens (human) | 7.37839E-08 | 7.132038 | ADCY9,AP2A2,AP2S1,ATP1A1,ATP1A4,KLK1,PLCB2,PLCB3,PRKACG,PRKCA,SLC8A1,VDR |
| hsa04672 | Intestinal immune network for IgA production - Homo sapiens (human) | 5.76982E-05 | 4.238838 | CD40,HLA-DMA,HLA-DQB1,HLA-DRA,HLA-DRB1,HLA-DRB3,HLA-DRB4,HLA-DRB5,IL6 |
| hsa04918 | Thyroid hormone synthesis - Homo sapiens (human) | 0.001398753 | 2.854259 | ADCY9,ASGR1,ATP1A1,ATP1A4,HSPA5,PLCB2,PLCB3,PRKACG,PRKCA |
| hsa04911 | Insulin secretion - Homo sapiens (human) | 0.003677303 | 2.434471 | ADCY9,ATP1A1,ATP1A4,PLCB2,PLCB3,PRKACG,PRKCA,RAB3A,STX1A |
| hsa05310 | Asthma - Homo sapiens (human) | 1.08523E-05 | 4.96448 | CD40,HLA-DMA,HLA-DQB1,HLA-DRA,HLA-DRB1,HLA-DRB3,HLA-DRB4,HLA-DRB5 |
| hsa05321 | Inflammatory bowel disease (IBD) - Homo sapiens (human) | 0.002370261 | 2.625204 | HLA-DMA,HLA-DQB1,HLA-DRA,HLA-DRB1,HLA-DRB3,HLA-DRB4,HLA-DRB5,IL6 |

*Pathways irrelevant to hematology and immunology were not shown in the table.
