# Supplementary material for: Circular RNA regulatory network reveals cell–cell crosstalk in acute myeloid leukemia extramedullary infiltration
Source: J Transl Med. 2018 Dec 17;16:361. doi: 10.1186/s12967-018-1726-x (PMC6297994; doi:10.1186/s12967-018-1726-x)
Supplement: Supplementary file 6 — Additional file 6: Table S6. KEGG pathway enrichment of downregulated genes between EMI and non-EMI AML samples. [file 12967_2018_1726_MOESM6_ESM.docx]

| **Table S6 KEGG pathway enrichment of downregulated genes between EMI and non-EMI AML samples** | | | | |
| --- | --- | --- | --- | --- |
| PathwayID | Definition | Fisher Pvalue | Enrichment Score | Genes |
| hsa04660 | T cell receptor signaling pathway - Homo sapiens (human) | 0.006711095 | 0.0375 | CD247,CD28,CD3D,CD40LG,ICOS,LCK,NCK1,PIK3R1,RASGRP1 |
| hsa05340 | Primary immunodeficiency - Homo sapiens (human) | 0.006874645 | 0.020833 | CD3D,CD40LG,ICOS,IGLL1,LCK |
| hsa04520 | Adherens junction - Homo sapiens (human) | 0.009233112 | 0.029167 | ACP1,CSNK2A1,LEF1,SORBS1,SSX2IP,WASL,YES1 |
| hsa00230 | Purine metabolism - Homo sapiens (human) | 0.01220897 | 0.05 | ADPRM,GMPR2,GUCY1B3,IMPDH2,NME7,NT5C3A,PDE5A,PNP,POLR1C,POLR1D,PRIM2,TWISTNB |
| hsa04672 | Intestinal immune network for IgA production - Homo sapiens (human) | 0.02188395 | 0.020833 | CCR9,CD28,CD40LG,ICOS,IL15RA |
| hsa01524 | Platinum drug resistance - Homo sapiens (human) | 0.03269554 | 0.025 | ATP7A,BCL2,CASP3,FASLG,PIK3R1,TOP2B |
| hsa05222 | Small cell lung cancer - Homo sapiens (human) | 0.03334918 | 0.029167 | BCL2,CASP3,CDK6,COL4A5,ITGA6,MAX,PIK3R1 |
| hsa00240 | Pyrimidine metabolism - Homo sapiens (human) | 0.04869671 | 0.029167 | NME7,NT5C3A,PNP,POLR1C,POLR1D,PRIM2,TWISTNB |
