# Supplementary material for: Circular RNA regulatory network reveals cell–cell crosstalk in acute myeloid leukemia extramedullary infiltration
Source: J Transl Med. 2018 Dec 17;16:361. doi: 10.1186/s12967-018-1726-x (PMC6297994; doi:10.1186/s12967-018-1726-x)
Supplement: Supplementary file 7 — Additional file 7: Figure S1. Regulatory networks of EMI-related circRNAs, miRNAs and genes. [file 12967_2018_1726_MOESM7_ESM.docx]

**Additional file 7: Figure S1 Regulatory networks of EMI-related circRNAs, miRNAs and genes.**

The overexpressed (A) and underexpressed (B) circRNA/miRNA/mRNA regulatory networks related to EMI were performed. Round nodes represent circRNAs, V nodes represent differentially expressed genes, red represents upregulated circRNAs and genes (A), and green represents downregulated circRNAs and genes (B). Blue triangles represent predicted miRNAs. The arrows indicate targeted regulatory relationships. Downregulated genes were not associated with specific EMI-related processes (Supplementary Table 5 and 6)
